# Elevational distribution of montane Afrotropical butterflies is influenced by seasonality and habitat structure

**DOI:** 10.1371/journal.pone.0270769

**Published:** 2022-07-05

**Authors:** Devolent T. Mtui, Joseph O. Ogutu, Raymond E. Okick, William D. Newmark

**Affiliations:** 1 Tanzania Wildlife Research Institute, Arusha, Tanzania; 2 Biostatistics Unit, Institute of Crop Science, University of Hohenheim, Stuttgart, Germany; 3 Natural History Museum of Utah, University of Utah, Salt Lake City, Utah, United States of America; Sikkim University, INDIA

## Abstract

Determinants of elevational distribution of butterfly species richness and abundance in the tropics are poorly understood. Here we assess the combined effects, both additive and interactive, of seasonality and habitat structure on the elevational distribution of butterflies in the Uluguru Mountains, Tanzania. We sampled butterflies along a 1100 m elevational gradient that extended from 1540 to 2639 m using a time-constrained fixed-area method during the short to long rains and long to short rains transitions, and in habitat structure classified as closed or open. We used semi-parametric generalized linear mixed models to assess the relation between butterfly species richness or abundance, and seasonality, habitat structure, family and elevation. For all species combined, species richness declined with elevation in both open and closed habitats during the long to short rains transition. During the short to long rains transition, species richness displayed a mid-elevation peak across habitats. Among the three focal families (Nymphalidae, Papilionidae and Pieridae) similar patterns in the elevational distribution of species richness were observed. Species abundance declined or remained stable with elevation across seasons and habitat structure; the exception being species abundance in open habitat during the short to long rain transition and increased slightly with elevation. Abundance by family did not vary significantly by habitat structure or season. Our results indicate that seasonality and habitat structure shape species richness and abundance of butterflies along an elevational gradient in the Uluguru Mountains. These patterns are important for informing conservation actions because temperature as well as annual and seasonal variation in precipitation are predicted to increase in East Africa as a result of climate change, important determinants of seasonality, while habitat disturbance may increase due to a projected doubling in Tanzania’s population over the next 27 years.

## Introduction

Elevational distribution patterns of species can provide valuable insights into interactions between organisms and environmental gradients processes driving those patterns [1], and knowledge of these patterns are important for the development of effective conservation strategies. For insects the more commonly observed patterns of species richness with an increase in elevation are a decrease, a low-elevation plateau, a low-elevation plateau with a mid-elevational peak, and a mid-elevation peak [1–4].

A number of factors have been proposed as drivers of elevational distribution of species. McCain and Grytnes [1] have grouped these factors into four major categories: climate, space, evolutionary history, and biotic processes. Climatic hypotheses relate patterns of species richness to variation in abiotic factors including precipitation, temperature, productivity, humidity, and cloud cover [1, 5, 6]. Spatial hypotheses include species-area relations and spatial constraints and their influence on the elevational distribution of species [1, 7, 8]. Evolutionary history hypotheses have included variation in speciation and extinction rates, clade age and phylogenetic conservatism as determinants of elevational patterns of species [1, 9]. Finally, biotic interactions include habitat complexity, disturbance, competition, and mutualism as drivers of elevational patterns of species [1, 10, 11].

Most studies of the elevational distribution of insects have been conducted in temperate regions. In contrast, comparatively fewer studies have been conducted in the tropics although the vast majority of species occur here. Yet, even among the better-studied insect taxa in the tropics such as butterflies [12, 13] there have been comparatively fewer studies in the tropics than in temperate regions that have examined determinants of elevational distribution of species.

Both abiotic and biotic factors influence species richness and abundance of tropical Lepidoptera along elevational gradients. Of these factors seasonality [14, 15] and habitat structure [16, 17] have been shown to influence elevational distributions of Afrotropical Lepidoptera species richness and abundance. However, the combined effects of seasonality and habitat structure and their interaction on the elevational distribution of butterflies have not been examined, as per out knowledge, in the tropics unlike in the subtropics [18, 19]. This is salient because the combined effects of climate change and human disturbance–two of the most important drivers of species loss worldwide–are increasingly altering and impacting seasonality and habitat structure at many sites throughout the tropics [20–22].

Seasonality is an important determinant of resource availability and activity patterns in Lepidoptera [23] and for tropical Lepidoptera is highly influenced by patterns of precipitation [14, 24–29]. However, as Maicher et al. [14] note most studies have ignored the influence of seasonality on the elevational distributions of tropical Lepidoptera. On Mount Cameroon in West Africa, Maicher et al. [14] recently reported a mid-elevational peak in butterfly species richness and abundance and that this peak shifted seasonally with precipitation.

Habitat structure has also been shown to influence elevational patterns of species richness and abundance in Afrotropical Lepidoptera [17, 30]. Patchiness of habitat can increase niche availability for specialized species, and thus alter species richness and abundance along an elevational gradient [31]. Additionally, habitat type and structure can limit movement of species that are habitat-specific and thus also alter species richness and abundance along an elevational gradient [1, 30, 31].

Here we examine the combined influence of seasonality and habitat structure on the elevational distribution of butterfly species richness and abundance in the Uluguru Mountains, an Afrotropical biodiversity hotspot. Specifically, we compare the influence of the short to long rains transition versus the long to short rains transition (season), closed versus open habitat structure, family (see Study groups in Methods) and their interactions on the elevational distribution of butterfly species richness and abundance. Based on associations reported from non-elevational studies of tropical butterfly species richness, abundance and seasonality and habitat structure [24–26, 28, 29] we hypothesize the following: Butterfly species richness and abundance in the Uluguru Mountains will be higher (1) during the short to long rains transition than during the long to short rains transition; (2) in open than in closed habitats; and (3) at lower than higher elevations.

## Materials and methods

### Study site

**Fig 1:**
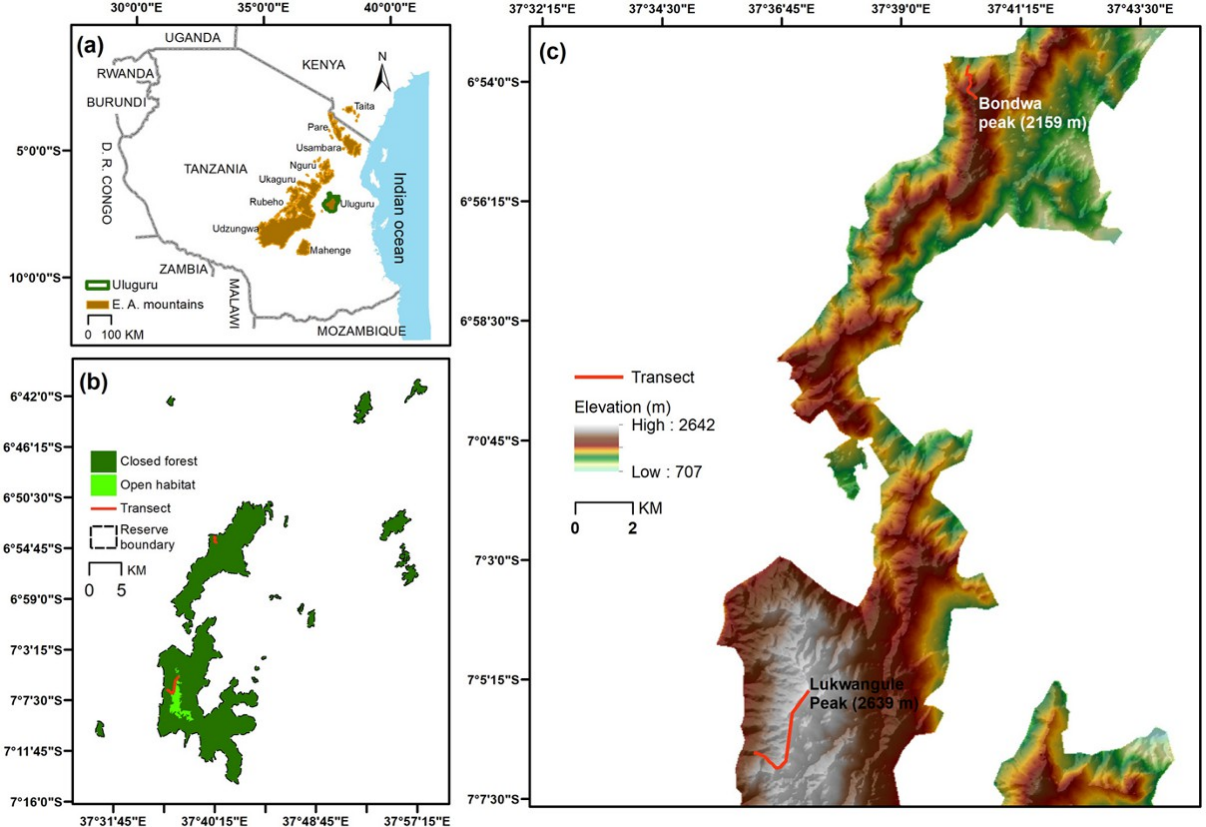
Map of the Uluguru Mountains illustrating (a) the location of the Eastern Arc Mountains including the Uluguru Mountains; (b) coarse-scale location of closed and open habitat and transects in the Uluguru Mountains; and (c) medium-scale location of transects in the Uluguru Mountains.

The study was conducted in the Uluguru Nature Reserve in east-central Tanzania (Fig 1). The Uluguru Mountains are part of the Eastern Arc Mountains that extend from the Taita Hills in south-east Kenya to the Udzungwa Mountains in south-central Tanzania [32–34]. The Eastern Arc Mountains are one of 36 global biodiversity hotspots [35] and have one of the highest ratios of endemic plant and animal species to area of any of the 36 biodiversity hotspots [36].

### Climatic data

**Fig 2:**
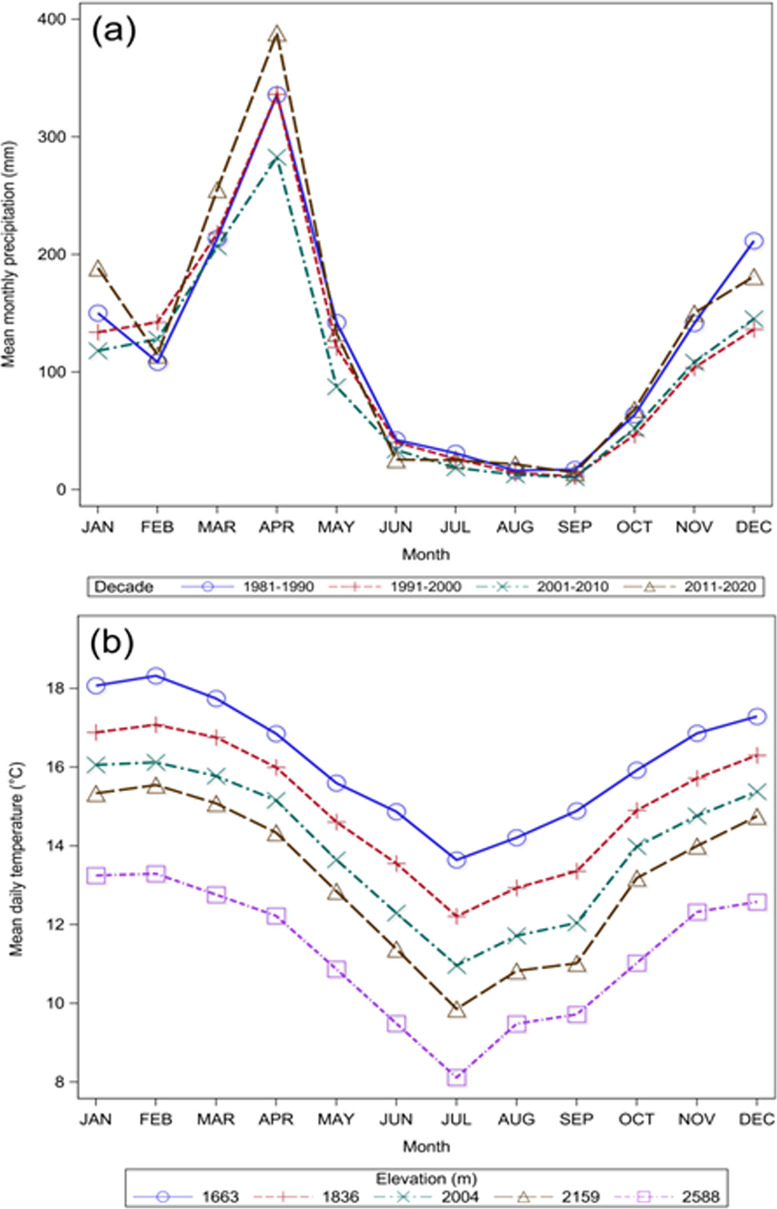
Climate data for the Uluguru Mountains. Panel (a) displays mean monthly precipitation between 1980–2020 extracted from the Climate Hazards Group InfraRed Precipitation with Station (CHIRPS) dataset [37], and (b) mean daily temperature by elevation that was recorded on-site.

Precipitation in the Uluguru Mountains is bimodal [37] with the long rains occurring between March and May and the short rains between October and December (Fig 2A). The short to long rains transition extends between January and February and the long to short rains transition extends between June and September (Fig 2A). Precipitation varies by aspect with higher annual totals along the eastern than the western side of the mountain. Mean annual precipitation along the eastern side of the mountain is approximately 2,400 mm [32]. The temporal duration of the short to long rains transition and long to short rains transition, however, does not vary by elevation in our study system. Mean monthly precipitation between 1980–2020 in the Uluguru Mountains was taken from the Climate Hazards Group InfraRed Precipitation with Station (CHIRPS) dataset [37] and are shown in Fig 2A.

Mean daily temperature also varies seasonally with the short to long rains transition coinciding with the hot season and the long to short rains transition coinciding with cool season (Fig 2B). The recorded lapse rate in the Uluguru Mountains is 0.55°C per 100 m (S1 Fig).

### Butterfly sampling

We conducted butterfly sampling along a 1100 m elevational gradient that extended from 1540 m to 2639 m (Fig 1B and 1C and S1 Table). This gradient represents the current elevational distribution of natural forest in the Uluguru Mountains. With the exception of a few small forest patches, all natural forest below 1540 m (300 m– 1540 m) has been cleared over the last 200–300 years by small-scale agriculture expansion [32, 33].

Sampling was conducted along two transects that followed established trails in the two largest forest blocks in the northern (1540–2159 m; 619 m change in elevation) and southern (2240–2639 m; 399 m change in elevation) sections of the Uluguru Nature Reserve (Fig 1). Thus, in our study system transect location and elevation are confounded, because sampling locations in the northern section of the Uluguru Mountains are located at lower elevations than sampling locations in the southern section of the Uluguru Mountains. To evaluate the effect of transect location on butterfly species richness and abundance independent of elevation, habitat structure, and season would require replicated elevational transects. However, cutting vegetation, which would be a requisite to establish additional transects, is prohibited in nature reserves in Tanzania.

We sampled butterflies along the two transects at an interval of ~ 40 m in elevation (range 30 m– 50 m). This interval was selected to permit a fine-scale assessment of the influence of seasonality and habitat structure on the elevational distribution of butterfly species. Prior to initiating sampling and to ensure an approximate equal difference in elevation between adjacent sampling locations, the location for all sampling locations along the transect was defined by waypoints (S1 Table) extracted from a digital elevation model of Tanzania. These pre-selected waypoints were used to locate and mark the position of 28 sampling locations along the two transects in the field. Sixteen sampling locations were located along the northern transect and twelve sampling locations were located along the southern transect (Fig 1 and S1 Table).

Between February 2019 and September 2020, we sampled butterflies at each of the 28 sampling locations on 20 occasions over two seasons. At each sampling location, 10 replicated samples were conducted during both the short to long rains transition (January through February) and long to short rains transition (June through September). We selected these seasons, which follow Maicher et al [14], to permit a direct comparison of phenological changes in butterfly community structure in montane forests in East Africa with montane forests in West Africa. Because of logistical constraints (road closures due to landslides) in accessing higher elevation sampling locations during the long rains, we did not conduct sampling during this time period.

At each sampling location along the transect, we sampled butterflies for 20 minutes in a 5 m × 5 m × 5 m plot centered over the trail through visual observations and sweep-net inspections of species we could not identify in the air following sampling protocols outlined in Caldas and Robbins [38]. We selected a 20-minute sampling period to enhance the likelihood of detecting rare species within a sampling plot. A team of five individuals (four observers and one recorder) conducted all sampling. Two observers identified butterflies visually and conducted sweep-net inspections of species, and the other two observers located and tracked individual butterflies in a sampling plot to reduce the likelihood of double-counting individuals. Post-hoc analysis indicates the mean (±SE) number of individuals per species in a sample was 0.25 ± 0.03, confirming the low-likelihood that individuals were double-counted. The number of visual identifications and sweep-net inspections of butterfly species varied between sampling locations and replicated samples due to the number of butterflies that was observed during a 20-minute time-constrained sample. Because of the small size of the sampling plot and the number of observers, we believe we were able to detect most butterfly species that occurred in a sampling plot during each sampling session with the exception of butterflies within the families Lycaenidae and Hesperiidae, which were excluded from the analysis (see Study groups in methods).

In addition, we placed a baited Van Someren trap [39] at each sampling location 24-hours prior to the sample. One baited trap was placed at the corner of each study plot, 2.5 m from the center of a trail, and were deployed on 20 occasions over two seasons. Traps were baited with fermented bananas [39]. During a 20-minute sample, all butterflies that were visually observed or identified in sweep-nets or in baited traps were recorded and voucher specimens collected. For species that could be easily identified in the field, voucher specimens were not collected. We identified butterflies species in the field using Kielland [12] and Martins and Collins [40]. Unidentified voucher specimens were taken to the African Butterfly Research Institute in Nairobi for identification. Total sampling effort across all sampling locations and seasons was 6905 sample hours.

### Habitat structure and vegetation

We classified habitat structure at each sampling location as either closed (i.e., forest) or open (i.e., non-forest) based on mean canopy closure. At each sampling location, we recorded four measurements of canopy closure using a densitometer [41] held at breast height, and located 1.5 m from the center of the 5 × 5 × 5 m sampling plot and facing in the four cardinal directions. We classified habitat structure as closed if the canopy closure was >50% or open if the canopy closure was ≤ 50% following Hansen et al [42] (S1 Table). Closed habitat was dominated by canopy-forming trees (see next paragraphs). Open habitat was dominated by low-stature vegetation comprised largely of bracken ferns, herbs, shrubs and grasses. In our study system, open habitat is largely a result of past human disturbance with historic cultivation (abandoned farmlands) being the dominant cause of disturbance at lower elevations and fire being an important cause of disturbance at higher elevations [43].

Pócs [43] has published a detailed description of the elevational distribution of vegetation through which the transect passed in the Uluguru Mountains. Forest between 1500–1800 m in the northern portion of the Uluguru Mountains and between 2000 and 2400 in the southern portion of the Uluguru Mountains (Fig 1B and 1C) are classified by Pócs [43] as mesophilous montane forest with an average canopy height of 20–30 m. The dominant tree species at these elevations are *Podocarpus milanjianus*, *Ocotea usambarensis*, *Afrocrania volkensii*, *Ficalhoa laurifola*, and *Cussonia spicata*. The common shrubs are *Mostuea brunonis*, *Chassalia parviflora*, *Chassalia violacea*, *Lasiodiscus usambarensis*, *Galineria coffeoides*, *Memecyclon mytrilloides*, *Erthrococca usambarica*, *Euphorbia usambarica*, and *Bridelia brideliffolia* while the common woody herbs include *Crassocephalum manni*, *Conyza newii*, *Vernonia adoensis* and *Ensete ulugurense* [43].

Between 1500–2300 m, sites we classified as open are dominated largely by non-canopy forming tree species and a combination of shrubs including *Mostuea brunonis*, *Chassalia parviflora*, *Chassalia violacea*, *Lasiodiscus usambarensis*, *Galineria coffeoides*, *Memecyclon mytrilloides*, *Erthrococca usambarica*, *Euphorbia usambarica*, and *Bridelia brideliffolia*, woody herbs include *Crassocephalum manni*, *Conyza newii*, *Vernonia adoensi*s and *Ensete ulugurense*, and grasses and herbs comprised of *Hyparrhenia rufa*, *H*. *diplandra*, *Brachiaria brizantha*, *Bekeropsis uniseta*, *Arthraxon quartinianus*, *Cleistachne sorghoides*, *Polygala macrostigma*, and *Habenaria splendens* [43].

Between 2300–2664 m in the southern Uluguru Mountains sites we classified as open are defined by Pócs [43] as moorland or subalpine grassland. In moorland, *Pycreus nigricans* is the dominant species and can reach heights of up to 5 m. In subalpine grassland the dominant species of grass are *Panicum lukwangulense*, *Andropogon amethystinus*, *Agrostis kilimandscharica*, and *Pteridium aquilinum*, and herbs and forbs are *Geranimum vagans*, *Helichrysum cymosum*, *H*. *abietinum*, *Senecio cyaneus*, *Lobelia holstii*, and *Blaeria johannnis* combined with a few scattered trees and shrubs comprising of *Myrica salicifolia*, *Adendocarpus manni*, and *Berberis holstii* [43].

### Study groups

Previous analyses of tropical butterfly species richness along elevational gradients have revealed variation in patterns of species richness among taxonomic families [44] and within tribes [13]. Consequently, we examine patterns of species richness and abundance by family as well as across all species combined. We restrict our analyses, however, to species within three families (Nymphalidae, Papilionidae and Pieridae). We excluded from the analysis butterflies in the families Lycaenidae and Hesperiidae because of the difficulty in accurately sampling these species due to their small body size and cryptic nature, and because in the tropics these families are taxonomically challenging [45]. In our study system many of these species occur in dense understory vegetation (grass, herbs, and forbs) and do not readily flush.

### Statistical analyses

The statistical analyses involved two response variables, species richness (number of species) and species abundance (number of individuals), which were regressed separately on four predictor variables: elevation, season, habitat structure, and family, and all their possible interactions. Season (short to long rains transition and long to short rains transition) and habitat structure (closed and open) each had two levels, while families had three levels (Nymphalidae, Papilionidae and Pieridae).

We first modelled the variation in species richness (number of species recorded within each 5 x 5 x 5 m plot per 246.6 sampling-hours) along an elevational gradient using a semi-parametric generalized linear mixed model (with a negative binomial error distribution and a log link function) and included habitat structure, and family, and all their interactions as factor-type covariates. The fixed part of the model consisted of parametric fixed effects whereas the random part contained non-parametric smoothed effects, hence the name semi-parametric model. The full model was fitted in the GLIMMIX Procedure in the SAS Software [46] using the restricted log-pseudo likelihood [47] and Fisher scoring in the first 10 iterations. The SAS code used to fit all the models is provided in the text in (S1 Text).

The parametric fixed effects were estimated for season, habitat structure, family and all their two- and three-way interactions. The random part of the mixed model consists of nine random effects or variance components. Of these, eight are continuous random effects that perform penalized spline smoothing of the distribution of species richness along elevation. The first captures patterns in the trend in species richness across the elevational gradient common to all the species records, the second to the seventh, respectively, capture patterns in the trend across the elevational gradient common to both seasons, both habitat types, all the three families, *seasons × habitats*, *seasons × families*, *habitats × families*, *seasons × habitats × families*. The ninth variance component is the scale (dispersion) parameter of the negative binomial distribution.

A tenth variance component was included to capture spatial autocorrelation in the residuals, represented in terms of the spatial generalization of the first-order autoregressive error structure and assuming an exponentially decaying correlation with distance of separation between observation points along the transect. But this component was not well supported by the data and resulted in an infinite likelihood, likely because it is confounded with the scale parameter of the negative binomial distribution. Thus, the model without spatially autocorrelated residuals adequately accounted for spatial autocorrelation.

The penalized cubic basis splines used with each continuous random effect has 10 equally spaced interior knots plus three knots at the start and three at the end of the observed values of elevation. Because the effect of *habitat × families* approximated significance, we decomposed this interaction into its simple effect slices. Additionally, the adjusted means and their 95% confidence limits and pairwise differences were tested for significance, after adjusting for multiplicity using simulation adjustment. Predicted species richness and its 95% confidence limits were back transformed to the original count scale using the inverse link (log) function. Residual diagnostics (Q-Q Plots, residual versus linear predictor plots, histograms and box-whisker plots for conditional raw residuals, conditional studentized residuals and conditional Pearson residuals) were used to check model fit, specifically the conditional raw residuals, conditional studentized residuals, and conditional Pearson residuals. The denominator degrees of freedom of the Wald-type F-tests of the fixed effects were adjusted for small sample size using the Kenward-Roger method [48]. The same modelling process was repeated for species abundance (number of individuals). Aggregate abundance for all the three families combined were similarly modelled after dropping the factor families from the models.

Due to the possibility of incomplete sampling of species richness, we estimated species richness with the abundance-based bias-corrected Chao1 estimator in Excel. Because observed and predicted species richness were highly correlated (Pearson correlation coefficient *r* = 0.91, P < 0.001, S2 Fig), we report results only for observed species richness. We selected Chao1 as an estimator of species richness because the abundance data contained both singleton and doubleton records, and for such data Chao1 is well-suited [49].

Research approval including permission to collect voucher specimens was provided by the Tanzania Wildlife Research Institute and Tanzania Commission on Science and Technology under permit number: 2020-435-NA-2018-259. Animal ethics were carefully considered during the collection of voucher specimens.

## Results

Across all samples and sampling locations, we recorded 1268 butterflies representing 56 species. [Nymphalidae: 472 butterflies, 41 species; Papilionidae: 222 butterflies, 7 species; and Pieridae: 574 butterflies, 8 species] (S2 and S3 Tables). During the seasonal transition between the short to long rains we recorded across all samples and sampling locations, 951 butterflies representing 48 species. [Nymphalidae: 302 butterflies, 33 species; Papilionidae: 83 butterflies, 7 species; and Pieridae 466 butterflies, 8 species]. During the seasonal transition between the long to short rains we recorded 317 butterflies representing 28 species [Nymphalidae: 170 butterflies, 22 species; Papilionidae: 39 butterflies, 4 species; and Pieridae: 108 butterflies, 2 species] (S4 Table).

Across all samples and sampling locations, we recorded in closed habitat 506 butterflies representing 25 species [Nymphalidae: 220 butterflies, 16 species; Papilionidae: 115 butterflies, 5 species; and Pieridae: 171 butterflies, 4 species]; and 762 butterflies representing 50 species in open habitat [Nymphalidae: 252 butterflies, 35 species; Papilionidae: 107 butterflies, 7 species; and Pieridae: 403 butterflies, 8 species] (S5 Table).

## Elevational patterns of butterfly species richness and abundance by habitat structure and season

### Species richness

**Fig 3:**
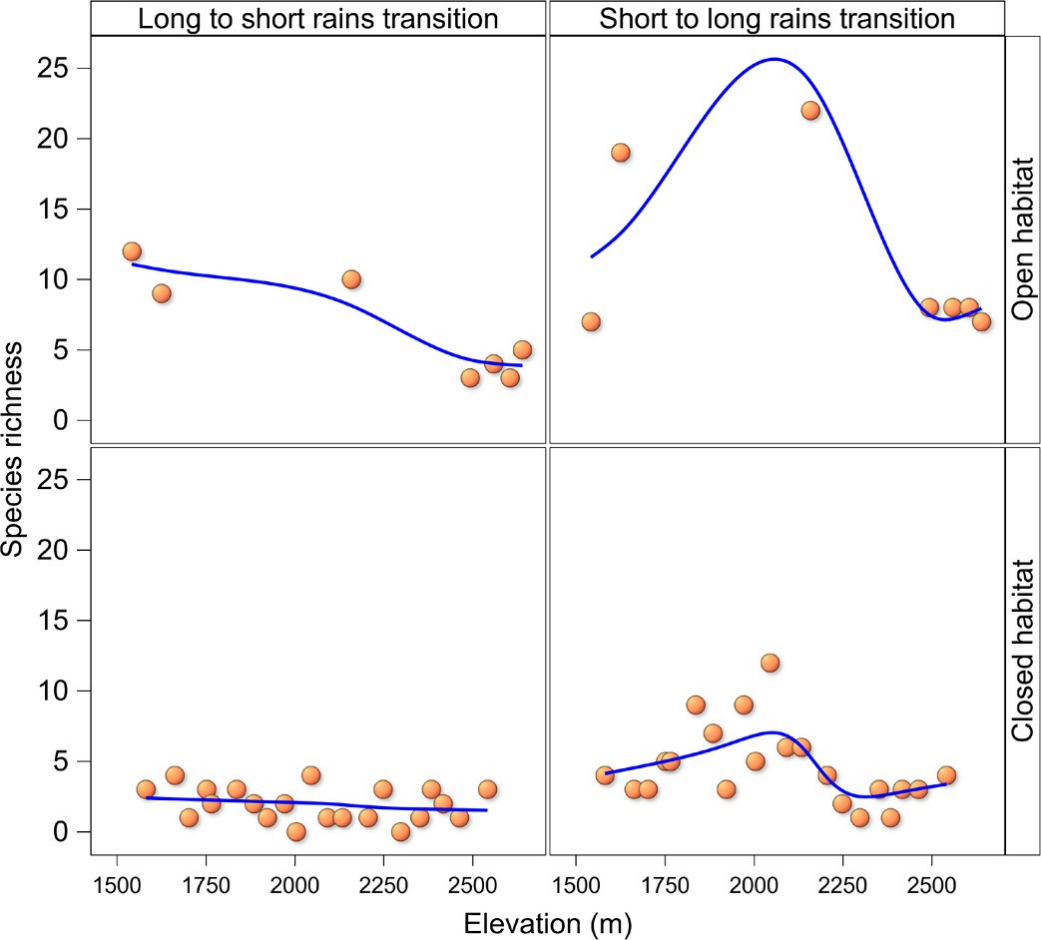
Patterns of species richness by season and habitat structure along an elevational gradient in the Uluguru Mountains. Trends lines were computed using Semi-parametric Generalized Linear Mixed Models.

Among all species combined, species richness decreased with elevation in both closed and open habitats during the long to short rains transition, but displayed a humped-shaped distribution with a mid-elevational peak at approximately 2050 m in elevation in closed and open habitats during the short to long rains transition (Fig 3). Among all species combined, species richness across the elevational gradient, was significantly higher (P <0.001) in open than in closed habitat. Total species richness was also significantly higher (P <0.004) during the short to long rains transition than during the long to short rains transition (Tables 1 and S6).

**Table 1:** Results of Semi-parametric Generalized Linear Mixed Models assessing the effects of elevation, season, habitat structure, and family on species richness and abundance along an elevational gradient in the Uluguru Mountains. NDF is the numerator and DDF is the denominator degrees of freedom for the F test. * p< 0.05, ** p< 0.01, *** p< 0.001.

Focal group	Effect	NDF	DDF	F Value	Prob F
Species richness	Season	2	4.34	27.72	0.003**
	Habitat Structure	1	37.85	43.34	<0.001***
	Season*Habitat Structure	1	37.85	0.404	0.529
	Elevation*Season*Habitat Structure	4	23.03	2.68	0.057
Species richness by family	Season	2	5.21	4.98	0.062
	Habitat Structure	1	18.35	6.66	0.019**
	Season*Habitat Structure	1	122.92	1.69	0.197
	Family	2	4.13	2.67	0.180
	Season*Family	2	7.25	0.72	0.521
	Family*Habitat Structure	2	24.90	5.04	0.015**
	Season*Family*Habitat Structure	2	122.28	0.21	0.809
	Elevation*Season*Family*Habitat Structure	12	33.13	1.89	0.072
Abundance	Season	2	4.03	44.30	0.002**
	Habitat Structure	1	4.67	8.10	0.039*
	Season*Habitat Structure	1	4.67	0.03	0.863
	Elevation*Season*Habitat Structure	4	4.77	0.371	0.820
Abundance by family	Season	2	3.67	5.28	0.083
	Habitat Structure	1	4.26	0.69	0.450
	Season*Habitat Structure	1	36.97	0.263	0.610
	Family	2	4.70	2.44	0.188
	Season*Family	2	2.58	0.19	0.836
	Family*Habitat Structure	2	4.09	2.78	0.172
	Season*Family*Habitat Structure	2	45.63	0.50	0.611
	Elevation*Season*Family*Habitat Structure	12	4.58	0.95	0.573

**Fig 4:**
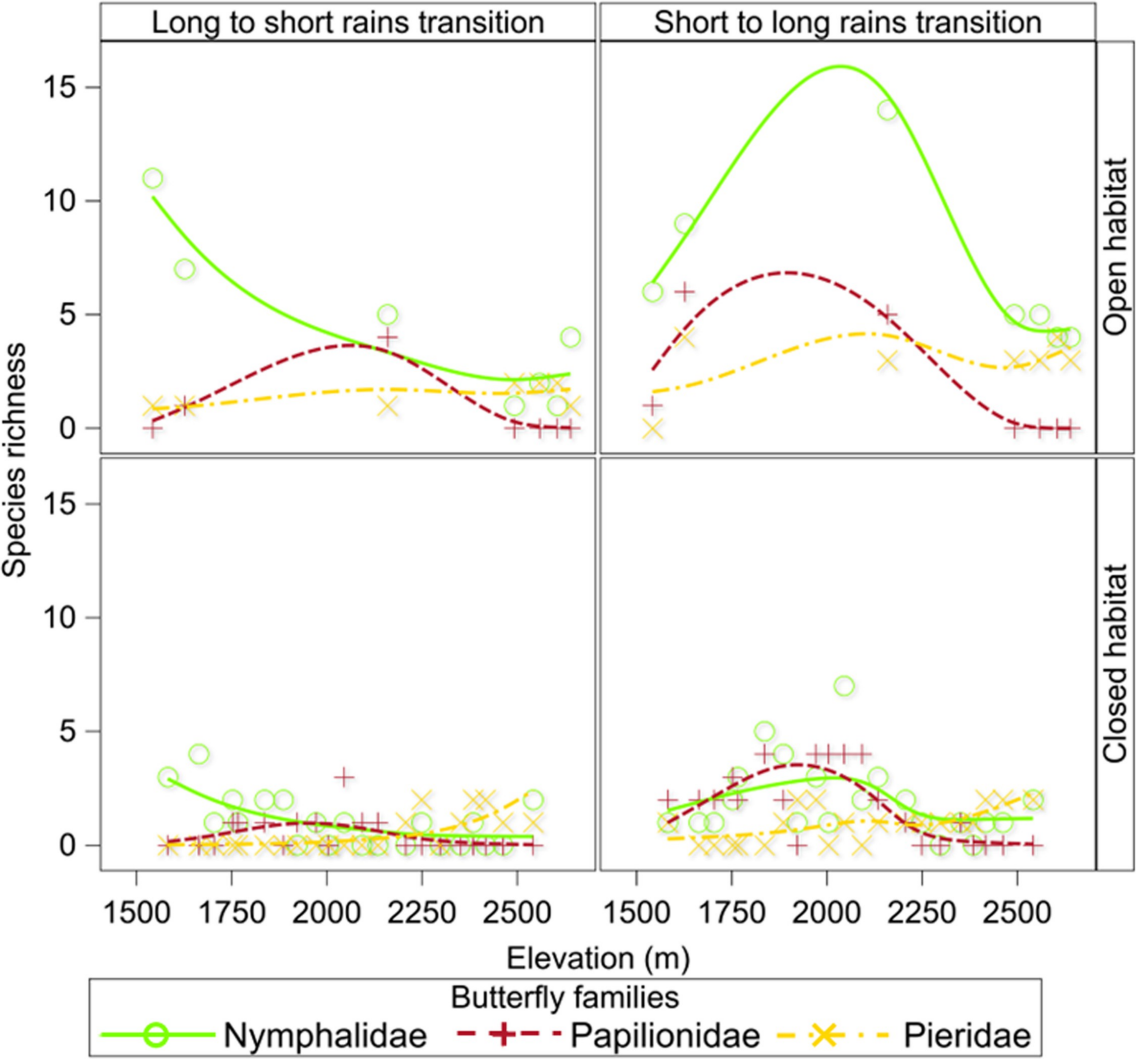
Patterns of species richness by family, season, and habitat structure along an elevational gradient in the Uluguru Mountains. Trends lines were computed using Semi-parametric Generalized Linear Mixed Models.

Across two of the three focal families (Papilionidae and Nymphalidae) species richness displayed a hump-shaped mid-elevational peak (~2000 m) in both closed and open habitats during the short to long rains transition while species richness in the family Pieridae increased with elevation in both closed and open habitats in both seasons (Fig 4). Butterfly species richness by family across the elevational gradient, was significantly higher (P <0.02) in open than in closed habitat but comparable (P >0.5) between seasons (Table 1). The interaction between family species richness and habitat structure (Tables 1 and S6) was also significant (P <0.02).

### Abundance

**Fig 5:**
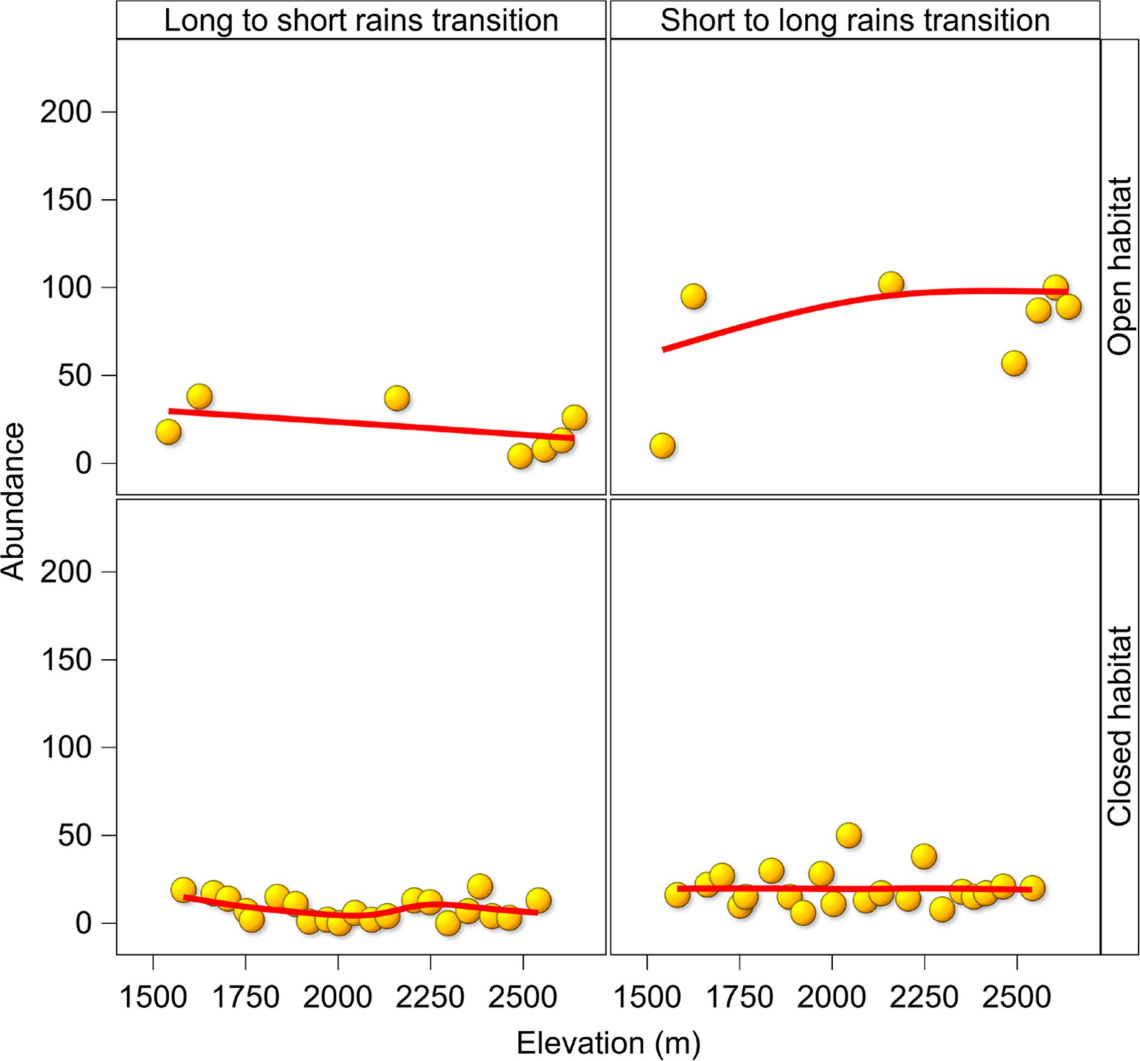
Patterns of butterfly abundance for all species combined by season and habitat structure along an elevational gradient in the Uluguru Mountains. Trends lines were computed using Semi-parametric Generalized Linear Mixed Models.

Butterfly abundance either declined or changed little along an elevational gradient by season and habitat structure (Fig 5). The exception being butterfly abundance in open habitat which increased slightly with elevation during the short to long rains transition. Butterfly abundance was significantly higher (P < 0.04) in open than in closed habitats, and during the short to long rains transition than during the long to short rains transition (P <0.003, Tables 1 and S6).

**Fig 6:**
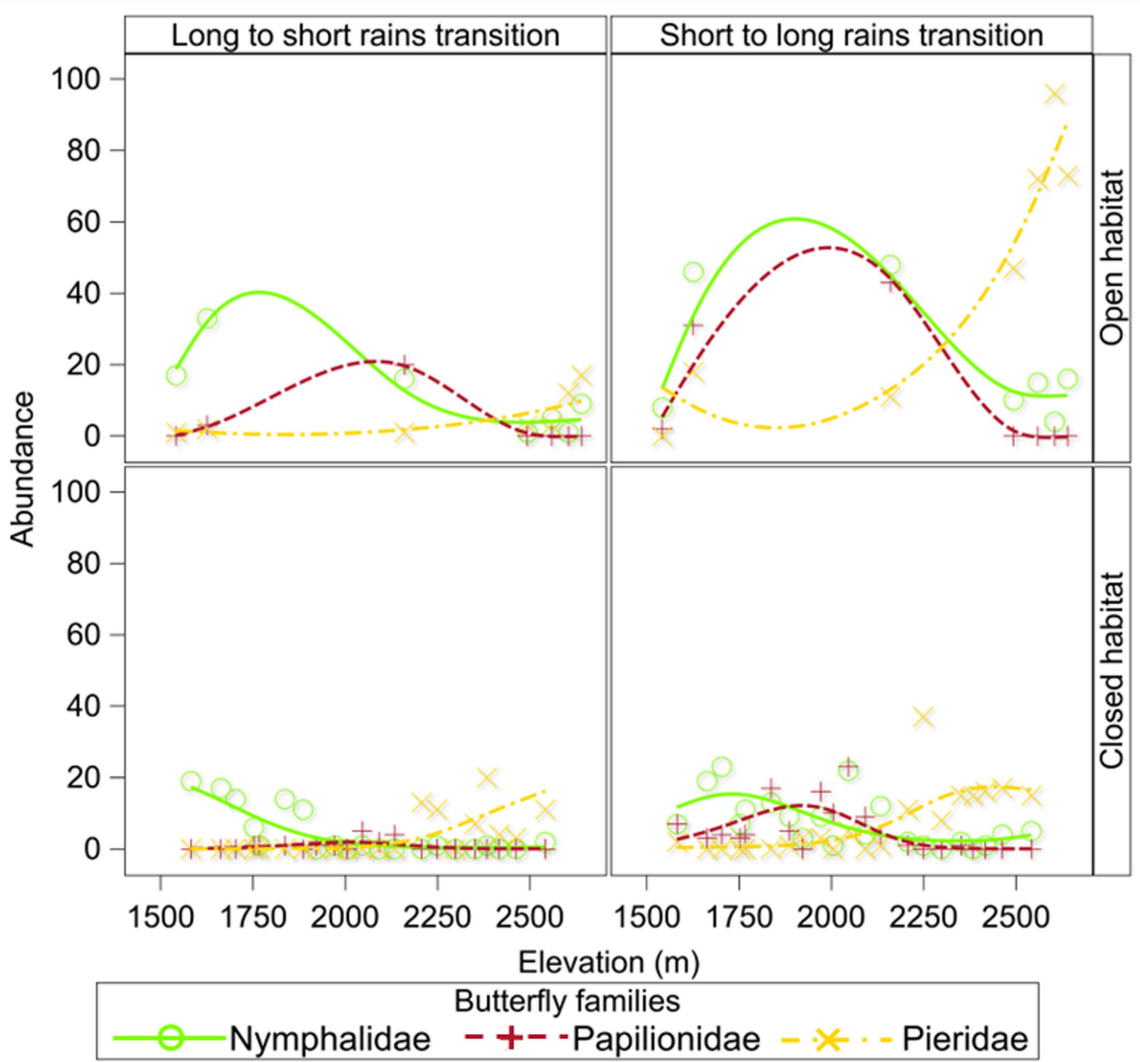
Patterns of butterfly abundance by family (Nymphalidae, Papilionidae, and Pieridae) and season and habitat structure along an elevational gradient in the Uluguru Mountains. Trends lines were computed using Semi-parametric Generalized Linear Mixed Models.

For two of the three families (Papilionidate and Nympalidae) the elevational distribution of butterfly abundance displayed a hump-shaped distribution at low to mid elevations (~ 1700 m– 2000 m) in open habitat during both the long to short rains and short to long rains transitions; and in closed habitat during the short to long rains transition (Fig 6). Butterfly abundance in the family Peiridae increased with elevation in both closed and open habitats and during both the short to long rains and long to short rains transitions. Butterfly abundance by family, however, did not vary significantly (P >0.05) by habitat, nor season (Tables 1 and S6).

## Discussion

Our results indicate that elevational patterns of butterfly species richness and abundance are influenced by both seasonality and habitat structure in the Uluguru Mountains. We observed higher species richness and abundance along an elevational gradient in the Uluguru Mountains during the short to long rains transition than during the long to short rains transition. We also observed higher species richness and abundance along the same elevational gradient in open than in closed habitat. However, we did not detect a significant interaction between season and habitat structure along an elevational transect for either butterfly species richness or abundance indicating that the effects of these predictor variables on butterfly species richness and abundance were additive rather than interactive along an elevational gradient in the Uluguru Mountains.

The influence of seasonality on elevational patterns of butterfly species richness and abundance in the Uluguru Mountains is consistent with findings from Mount Cameroon in west Africa [14]. Maicher et al. [14] reported higher butterfly species richness and abundance during the dry season relative to either the wet to dry season transition or dry to wet season transition. However, comparing the precise temporal peak in butterfly species richness and abundance in the Uluguru Mountains with Mount Cameroon is difficult because of differences in the seasonal timing and amount of precipitation received. Mount Cameroon receives five times the annual precipitation of the Uluguru Mountains. However, in both Uluguru Mountains and on Mount Cameroon annual temporal peak in butterfly species richness and abundance is associated with the “dry season”. Finally, our results support conclusions by Maicher et al. [14] about the importance of considering seasonality when interpreting elevational patterns of tropical Lepidoptera.

The seasonal peak in butterfly species richness and abundance in the Uluguru Mountains during the short to long rains transition is also consistent with seasonal patterns of butterfly species richness and abundance in the Kibale forest in Uganda although the annual timing of this seasonal peak differs between these two locations. In the mid-elevation (1100 m– 1590 m) Kibale forest, long-term monitoring of butterflies over a 132 month-period revealed bi-annual peaks in species richness and abundance that occur 3–4 months after the peak in the short (termed “small” rains) and long rains (termed “large” rains) and corresponding to 2–3 months after the peak in vegetation greenness. However, in the Kibale forest the “small” rains occur between March-May, which coincides temporally with the long rains in the Uluguru Mountains. While the “large” rains in Kibale forest occur between September–November, which coincides temporally with the short rains in the Uluguru Mountains. Yet, both in the Uluguru Mountains and to a lesser degree in the Kibale forest butterfly species richness and abundance is higher during the short to long rains transition than during the long to short rains transition (see Fig 1 in Valtonen et al. 2013) [50].

One possible explanation for the seasonal peak in diversity and abundance of butterflies in the Uluguru Mountains during the short to long rains seasonal transition may possibly relate to the seasonal timing of host plant greenness for larvae [50]. Consequently, identifying host and food plant phenology in the Uluguru Mountains should be a priority for future research. An alternative and non-mutually exclusive explanation for the seasonal peak in butterfly diversity and abundance along an elevational gradient during the short to long rains transition in the Uluguru Mountains is, as Maicher et al. [14] have hypothesized, a result of variation in the phenological emergence of adults along an elevational gradient–due possibly to plant phenology, avoidance of predators and parasites, or both [51–53]. Finally, a seasonal peak in butterfly diversity and abundance along an elevational gradient during the short to long rains transition in the Uluguru Mountains may be a result of seasonal upslope and downslope movement of species. Seasonal movements of Lepidoptera have been observed along elevational gradients in montane habitats in Costa Rica [54, 55].

Habitat structure has also been shown to influence elevational and non-elevational patterns of species richness and abundance of Lepidoptera in previous studies in the Afrotropics [17, 30, 56]. Along an elevational gradient on Mount Kilimanjaro Axmacher & Fiedler (2008) [57] reported that geometrid moth diversity (Fisher’s α) was higher in open than closed habitats, which is consistent with our findings from the Uluguru Mountains. Maicher et al. [17] also have recently reported a significant interaction between elevation and elephant-caused habitat disturbance on Mount Cameroon with higher butterfly species richness in more disturbed or open sites on Mount Cameroon at lower (1100 m) elevations than at higher (1850 m) elevations. We hypothesize that higher butterfly species richness and abundance in open than in closed habitats along an elevational gradient in the Uluguru Mountains may be a result of higher light intensity and thus abundance of food resources and/or host plants for larval and adult stages. Assessing food resource abundance within closed versus open habitat along an elevational gradient in the Uluguru Mountains should also be a priority for future research.

In the Uluguru Mountains, patterns of species richness varied by family. Species richness for two of the three focal families (Papilionidae and Nymphalidae) displayed a hump-shaped mid-elevational peak at ~2000 m in both closed and open habitats during the short to long rains transition while species richness in the family Pieridae increased with elevation in both closed and open habitats in both seasons. On the other hand, butterfly abundance by family did not vary by habitat nor season.

Elevational patterns of species richness of butterfly families in the Uluguru Mountains also differ from that reported for these same families along an elevational transect that extended from 117 m to 3104 m in the Sierra de Juárez in southern Mexico [44] where a low-elevation plateau with a decline at upper elevations was observed for nymphalids; a decline with elevation for papilionids; and a mid-elevational peak for pierids. Elevational patterns of butterfly families in the Uluguru Mountains also differ from that reported in the subtropical Eastern Himalayas [18, 19]. These differences in elevational patterns of species richness both among families within the Uluguru Mountains and between families in the Uluguru Mountains and the Sierra de Juárez and the Eastern Himalayas may be a result of multiple factors. These include but are not limited to differences in the elevational extent of forest among these locations [1], physiological limits of species [1], abundance and diversity of predators and parasites [58], evolutionary histories [59–61], and climate-landuse interactions [62].

Over the last two centuries 77% of the original forest cover in the Eastern Arc Mountains have been lost [33]. The remaining forest in the 13 Eastern Arc Mountains is highly fragmented and is comprised of 311 fragments >10 ha in size with a median fragment size of 84 ha [33]. In addition, fires, logging, and firewood collection have also altered the habitat structure of the Eastern Arc forests in many regions over the last two centuries and particularly in regions in close proximity to human populations [32]. Over the last 35 years, Tanzania’s population has approximately tripled from 23 million in 1987 to more than 61 million today and is projected to double again over the next 27 years [63]. Human pressures on the Eastern Arc forests will almost certainly continue to grow over time, and particularly in forests where forest protection and management are minimal. Understanding the impact of forest disturbance on habitat structure is therefore critical for the conservation of biodiversity in the Eastern Arc Mountains and for developing effective conservation strategies in species and endemic rich biodiversity hotspots such as the Uluguru Mountains.

Global climate change is predicted to result in upslope shifts in the elevational range of many species [64]. Increased annual mean temperature over the last three decades in other nearby Eastern Arc Mountains (East and West Usambara Mountains) has been associated with upslope elevational range shifts and range contractions [65] as well as reductions in demographic rates [66] of understory bird species. Annual and seasonal variance in precipitation has also increased over the last five decades in East Africa [67]. Ectothermic organisms such as butterflies in the Uluguru Mountains may be particularly sensitive to future annual and seasonal changes in precipitation and temperature as evidenced by elevational range shifts in Lepidoptera on Mount Kinabalu in Borneo [68, 69]. Our results provide an important baseline to assess the impact of climate change and habitat degradation on the elevational distribution and abundance of butterflies in the Eastern Arc Mountains.

## Supporting information

S1 Data Datafile. (XLSX)

S1 Text SAS code. (DOCX)

S1 Table Description of sampling locations sites along two elevational transects in the Uluguru Mountains. (DOCX)

S2 Table Family and species by elevation. (XLSX)

S3 Table Family and species by method and elevation. (XLSX)

S4 Table Family and species by season and elevation. (XLSX)

S5 Table Family and species by habitat structure and elevation. (XLSX)

S6 Table Parameter estimates for semi-parametric generalized linear mixed models. (DOCX)

S1 Fig Lapse rate in the Uluguru Mountains. Air temperature was recorded with loggers at 1-h intervals at 1663, 1836, 2004, 2159, and 2588 m over a 14-month period between August 2019 and September 2020. The straight line is described by the following equation: y = 25.1–0.0055 (x).
(TIF)

S2 Fig The relation between observed and predicted species richness. Predicted species richness was calculated with the abundance-based bias-corrected Chao1 estimator.
(TIF)
