# Supplementary material for: Elevational distribution of montane Afrotropical butterflies is influenced by seasonality and habitat structure
Source: PLoS One. 2022 Jul 5;17(7):e0270769. doi: 10.1371/journal.pone.0270769 (PMC9255748; doi:10.1371/journal.pone.0270769)
Supplement: S1 Text — (DOCX) [file pone.0270769.s002.docx]

**Supporting Information**

**Text**

**SAS (base SAS Version 9.4, SAS/STAT Version 15.1) code used to fit models.**

**PROC** **IMPORT** OUT= WORK.Abundance

DATAFILE= "D:\joseph_2009\Devolent\Butterflies\Revised data with canopy cover_18Sept21\data for Joseph_29June21\abundance by season_3families_June21.xlsx"

DBMS=EXCEL REPLACE;

RANGE="Sheet1$";

GETNAMES=YES;

MIXED=NO;

SCANTEXT=YES;

USEDATE=YES;

SCANTIME=YES;

**RUN**;

**data** abundance2; set abundance;

array xx1[*] Nymphalidae--Pieridae;

do f=**1** to dim(xx1);

count=xx1[f];

select(f);

when (**1**) Families="Nymphalidae ";

when (**2**) Families="Papilionidae";

when (**3**) Families="Pieridae";

otherwise;

end;

If Canopyclosure_perc<=**50** then Canopy="Open ";

else if Canopyclosure_perc>**50** then Canopy="Closed";

output ;

end;

**run**;

ods output covparms=covparms_abund tests3=tests3_abund tests1=tests1_abund slicelines=slicelines_abund

slices=slices_abund slicediffs=slicediffs_abund Dimensions=Dimensions_abund ClassLevels=ClassLevels_abund

lsmeans=Lsmeans_abund diffs=diffs_abund Parameterestimates=Parameterestimates_abund;

**Proc** **glimmix** data= abundance2 method=RSPL initglm itdetails scoring=**10** plots=all;

class Season Families Canopy;

Model Count=Season|Canopy|Families Season*Canopy*Families*elevation /noint dist=negbin link=log ddfm=KR htype=**1**,**3** solution cl;

Random elevation /sub=intercept type=pspline(difforder=**3** degree=**3**) knotmethod=equal(**10**) ;

Random elevation /sub=Season type=pspline(difforder=**3** degree=**3**) knotmethod=equal (**10**) ;

Random elevation /sub=Canopy type=pspline(difforder=**3** degree=**3**) knotmethod=equal (**10**) ;

Random elevation /sub=Families type=pspline(difforder=**3** degree=**3**) knotmethod=equal (**10**) ;

Random elevation /sub=Season*Canopy type=pspline(difforder=**3** degree=**3**) knotmethod=equal (**10**) ;

Random elevation /sub=Season*Families type=pspline(difforder=**3** degree=**3**) knotmethod=equal (**10**) ;

Random elevation /sub=Canopy*Families type=pspline(difforder=**3** degree=**3**) knotmethod=equal (**10**) ;

Random elevation /sub=Season*Canopy*Families type=pspline(difforder=**3** degree=**3**) knotmethod=equal (**10**) ;

*Random _residual_ //*sub=Location*/ type=sp(exp)(lat long);

parms (**0**)(**0**)(**0**)(**0.043932141**)(**0**)(**0.000542706**)(**0.005076689**)(**0**)(**0.630160914**);

slice Families*Canopy/sliceby=Canopy adjust=simulate lines;

slice Families*Canopy/sliceby=Families adjust=simulate lines;

Lsmeans Families*Canopy/ slice=Canopy lines mean cl adjust=simulate;

Lsmeans Families*Canopy/slice=Families lines mean cl adjust=simulate;

output out=Pred_butterflies_abund Pred(ilink)=mu lcl(ilink)=lower ucl(ilink)=upper;

nloptions tech=NRRIDG Maxiter=**1000** maxfunc=**5000**;

run;

**data** myattrmap;

length Value $**20** linecolor $ **20** fillcolor $ **20**;

input ID $ value $ & linecolor $ & fillcolor $ &;

datalines;

myid Nymphalidae chartreuse chartreuse

myid Papilionida salmon salmon

myid Pieridae gold gold

;

**run**;

**data** Pred_butterflies_abund2; set Pred_butterflies_abund;

if Season="cold" then Season2="Long to short rains transition";

if Season="warm" then Season2="Short to long rains transition";

if Canopy="Open" then Canopy2="Open habitat ";

if Canopy="Closed" then Canopy2="Closed habitat";

**run**;

ods graphics on / imagename='Figure_abund' width=**28** cm height=**26** cm;

ods listing image_dpi=**300**;

**proc** **sgpanel** data= Pred_butterflies_abund2 dattrmap=myattrmap noautolegend;

Panelby Season2 Canopy2 /novarname layout= lattice columns=**2** rows=**2** spacing=**2** uniscale=all sort=data

HEADERATTRS=(Color=black Family=Arial Size=**12** Style=normal Weight=normal);

Scatter x=elevation y=Count / markerattrs=(size=**20**) group=Families Groupdisplay=overlay grouporder=data dataskin=Sheen attrid=myid name="name1" ;

Pbspline x=elevation y=mu / group=Families Lineattrs=(/*color=red pattern=1*/ thickness=**3**) nomarkers maxpoints=**100** nknots=**50** degree=**2** attrid=myid name="name2";

title height=**12** pt color=black "Abundance of three buftterfly families in cold and warm seasons and open and closed habitats";

colaxis label='Elevation (m)' Discreteorder=Data valuesrotate=diagonal LABELATTRS=(Color=black Family=Arial Size=**14**

Style=normal Weight=normal) VALUEATTRS=(Color=black Family=Arial Size=**10** Style=normal Weight=normal) ;

rowaxis label='Abundance' LABELATTRS=(Color=black Family=Arial Size=**12** Style=normal Weight=normal)

VALUEATTRS=(Color=black Family=Arial Size=**12** Style=normal Weight=normal);

Keylegend "name1" "name2"/ Position=bottom title= 'Butterfly families' autoitemsize VALUEATTRS=(family=Arial size=**12**) across=**3** down=**2** titleattrs=(color=black Family=Arial Size=**12**);

**run**;

**data** Abundance3; set Abundance;

If Canopyclosure_perc<=**50** then Canopy="Open ";

else if Canopyclosure_perc>**50** then Canopy="Closed";

if Season="cold" then Season2="Long to short rains transition";

if Season="warm" then Season2="Short to long rains transition";

if Canopy="Open" then Canopy2="Open habitat ";

if Canopy="Closed" then Canopy2="Closed habitat";

**run**;

ods output covparms=covparms_total_abund tests3=tests3_total_abund tests1=tests1_total_abund slicelines=slicelines_total_abund

slices=slices_total_abund slicediffs=slicediffs_total_abund Dimensions=Dimensions_total_abund ClassLevels=ClassLevels_total_abund

lsmeans=Lsmeans_total_abund diffs=diffs_total_abund Parameterestimates=Parameterestimates_total_abund;

**Proc** **glimmix** data= abundance3 method=RSPL initglm itdetails scoring=**10** plots=all;

class Season Canopy;

Model abund=Season|Canopy Season*Canopy*elevation /noint dist=negbin link=log ddfm=KR htype=**1**,**3** solution cl;

Random elevation /sub=intercept type=pspline(difforder=**3** degree=**3**) knotmethod=equal(**10**) ;

Random elevation /sub=Season type=pspline(difforder=**3** degree=**3**) knotmethod=equal (**10**) ;

Random elevation /sub=Canopy type=pspline(difforder=**3** degree=**3**) knotmethod=equal (**10**) ;

Random elevation /sub=Season*Canopy type=pspline(difforder=**3** degree=**3**) knotmethod=equal (**10**) ;

*Random _residual_ //*sub=Location*/ type=sp(exp)(lat long);

parms (**0**)(**0**)(**0**)(**0.000448**)(**0.3417**) ;

slice Season*Canopy/sliceby=Canopy adjust=simulate lines;

slice Season*Canopy/sliceby=Season adjust=simulate lines;

Lsmeans Season*Canopy/ slice=Canopy lines mean cl adjust=simulate;

Lsmeans Season*Canopy/slice=Season lines mean cl adjust=simulate;

output out=Pred_butterflies_total_abund Pred(ilink)=mu lcl(ilink)=lower ucl(ilink)=upper;

nloptions tech=NRRIDG Maxiter=**1000** maxfunc=**5000**;

run;

ods graphics on / imagename='Figure_total_abund'

width=**28** cm height=**26** cm;

ods listing image_dpi=**300**;

**proc** **sgpanel** data=Pred_butterflies_total_abund noautolegend;

Panelby Season2 Canopy2 /novarname layout= lattice columns=**2** rows=**2** spacing=**2** uniscale=all sort=data

HEADERATTRS=(Color=black Family=Arial Size=**18** Style=normal weight=normal);

Scatter x=elevation y=abund / markerattrs=(symbol=circlefilled size=**25** color=Orange) dataskin=Sheen /*seglabel */ name="name1" ;

Pbspline x=elevation y=mu / Lineattrs=(color=red pattern=**1** thickness=**5**) nomarkers maxpoints=**75** nknots=**20** degree=**2** /*seglabel */

name="name2";

*title height=18 pt color=black "Abundance of all butterfly families";

colaxis label='Elevation (m)' Discreteorder=Data valuesrotate=diagonal LABELATTRS=(Color=black Family=Arial Size=**18**

Style=normal weight=normal) VALUEATTRS=(Color=black Family=Arial Size=**18** Style=normal weight=normal) ;

rowaxis label='Abundance' LABELATTRS=(Color=black Family=Arial Size=**18** Style=normal weight=normal)

VALUEATTRS=(Color=black Family=Arial Size=**18** Style=normal weight=normal);

*Keylegend "name1" "name2"/ Position=bottom title= '' autoitemsize VALUEATTRS=(family=Arial size=12) across=3 down=2 titleattrs=(color=black Family=Arial Size=12);

*colaxistable Count/Class=Habitat Position=bottom Separator valueattrs=(color=black family=Helvetica size=10)

LABELATTRS=(Color=black Family=Arial Size=11 Style=normal Weight=normal) /*title="Habitat type"*/ ;

/*valueattrs=(color=red)

labelattrs=(color=red)

titleattrs=(color=red);*/

**run**;

**PROC** **IMPORT** OUT= WORK.Richness

DATAFILE= "D:\joseph_2009\Devolent\Butterflies\Revised data with canopy cover_18Sept21\data for Joseph_29June21\richness by season_3families_June21.xlsx"

DBMS=EXCEL REPLACE;

RANGE="Sheet1$";

GETNAMES=YES;

MIXED=NO;

SCANTEXT=YES;

USEDATE=YES;

SCANTIME=YES;

**RUN**;

**data** Richness2; set Richness;

array xx2[*] Nymphalidae--Pieridae;

do f=**1** to dim(xx2);

Richness=xx2[f];

select(f);

when (**1**) Families="Nymphalidae ";

when (**2**) Families="Papilionidae";

when (**3**) Families="Pieridae";

otherwise;

end;

If Canopyclosure_perc<=**50** then Canopy="Open ";

else if Canopyclosure_perc>**50** then Canopy="Closed";

count=**1**;

output ;

end;

**run**;

**proc** **means** data=Richness2 nway noprint;

class Season Families Canopy;

var count;

output out=Richness_counts(drop=_:) sum=;

**run**;

ods output covparms=covparms_rich tests3=tests3_rich tests1=tests1_rich slicelines=slicelines_rich

slices=slices_rich slicediffs=slicediffs_rich Dimensions=Dimensions_rich ClassLevels=ClassLevels_rich

lsmeans=Lsmeans_rich diffs=diffs_rich Parameterestimates=Parameterestimates_rich;

**Proc** **glimmix** data= Richness2 method=RSPL initglm itdetails scoring=**10** plots=all;

class Season Families Canopy;

Model Richness=Season|Canopy|Families Season*Canopy*Families*elevation /noint dist=negbin link=log ddfm=KR htype=**1**,**3** solution cl;

Random elevation /sub=intercept type=pspline(difforder=**3** degree=**3**) knotmethod=equal(**10**) ;

Random elevation /sub=Season type=pspline(difforder=**3** degree=**3**) knotmethod=equal (**10**) ;

Random elevation /sub=Canopy type=pspline(difforder=**3** degree=**3**) knotmethod=equal (**10**) ;

Random elevation /sub=Families type=pspline(difforder=**3** degree=**3**) knotmethod=equal (**10**) ;

Random elevation /sub=Season*Canopy type=pspline(difforder=**3** degree=**3**) knotmethod=equal (**10**) ;

Random elevation /sub=Season*Families type=pspline(difforder=**3** degree=**3**) knotmethod=equal (**10**) ;

Random elevation /sub=Canopy*Families type=pspline(difforder=**3** degree=**3**) knotmethod=equal (**10**) ;

Random elevation /sub=Season*Canopy*Families type=pspline(difforder=**3** degree=**3**) knotmethod=equal (**10**) ;

*Random _residual_ /sub=Location type=sp(exp)(lat long);

parms (**0.000137**)(**0**)(**0**)(**0.004719**)(**0**)(**0.000689**)(**0.000144**)(**0**)(**0.03675**);

slice Families*Canopy/sliceby=Canopy adjust=simulate lines;

slice Families*Canopy/sliceby=Families adjust=simulate lines;

Lsmeans Families*Canopy/ slice=Canopy lines mean cl adjust=simulate;

Lsmeans Families*Canopy/slice=Families lines mean cl adjust=simulate;

output out=Pred_butterflies_rich Pred(ilink)=mu lcl(ilink)=lower ucl(ilink)=upper;

nloptions tech=NRRIDG Maxiter=**1000** maxfunc=**5000**;

run;

**data** Pred_butterflies_rich2; set Pred_butterflies_rich;

if Season="cold" then Season2="Long to short rains transition";

if Season="warm" then Season2="Short to long rains transition";

if Canopy="Open" then Canopy2="Open habitat ";

if Canopy="Closed" then Canopy2="Closed habitat";

**run**;

ods graphics on / imagename='Figure_richness'

width=**28** cm height=**26** cm;

ods listing image_dpi=**300**;

**proc** **sgpanel** data= Pred_butterflies_rich2 dattrmap=myattrmap noautolegend;

Panelby Season2 Canopy2 /novarname layout= lattice columns=**2** rows=**2** spacing=**2** uniscale=all sort=data

HEADERATTRS=(Color=black Family=Arial Size=**12** Style=normal Weight=normal);

Scatter x=elevation y=Richness / markerattrs=(size=**20**) group=Families Groupdisplay=overlay grouporder=data dataskin=Sheen attrid=myid name="name1";

Pbspline x=elevation y=mu / group=Families Lineattrs=(/*color=red attern=1*/ thickness=**3**) nomarkers maxpoints=**100** nknots=**50** degree=**2** attrid=myid name="name2";

title height=**12** pt color=black "Species richness of three butterfly families in cold and warm seasons and open and closed habitats";

colaxis label='Elevation (m)' Discreteorder=Data valuesrotate=diagonal LABELATTRS=(Color=black Family=Arial Size=**14**

Style=normal Weight=normal) VALUEATTRS=(Color=black Family=Arial Size=**10** Style=normal Weight=normal) ;

rowaxis label='Species richness' LABELATTRS=(Color=black Family=Arial Size=**12** Style=normal Weight=normal)

VALUEATTRS=(Color=black Family=Arial Size=**12** Style=normal Weight=normal);

Keylegend "name1" "name2"/Position=bottom title= 'Butterfly families' autoitemsize VALUEATTRS=(family=Arial size=**12**) across=**3** down=**2** titleattrs=(color=black Family=Arial Size=**12**);

**data** Richness3; set Richness;

If Canopyclosure_perc<=**50** then Canopy="Open ";

else if Canopyclosure_perc>**50** then Canopy="Closed";

if Season="cold" then Season2="Long to short rains transition";

if Season="warm" then Season2="Short to long rains transition";

if Canopy="Open" then Canopy2="Open habitat ";

if Canopy="Closed" then Canopy2="Closed habitat";

**run**;

ods output covparms=covparms_total_Richness tests3=tests3_total_Richness tests1=tests1_total_Richness slicelines=slicelines_total_Richness

slices=slices_total_Richness slicediffs=slicediffs_total_Richness Dimensions=Dimensions_total_Richness ClassLevels=ClassLevels_total_Richness

lsmeans=Lsmeans_total_Richness diffs=diffs_total_Richness Parameterestimates=Parameterestimates_total_rich;

**Proc** **glimmix** data= Richness3 method=RSPL initglm itdetails scoring=**10** plots=all;

class Season Canopy;

Model Sprichness=Season|Canopy Season*Canopy*elevation /noint dist=negbin link=log ddfm=KR htype=**1**,**3** solution cl;

Random elevation /sub=intercept type=pspline(difforder=**3** degree=**3**) knotmethod=equal(**10**) ;

Random elevation /sub=Season type=pspline(difforder=**3** degree=**3**) knotmethod=equal (**10**) ;

Random elevation /sub=Canopy type=pspline(difforder=**3** degree=**3**) knotmethod=equal (**10**) ;

Random elevation /sub=Season*Canopy type=pspline(difforder=**3** degree=**3**) knotmethod=equal (**10**) ;

*Random _residual_ //*sub=Location*/ type=sp(exp)(lat long);

parms (**0**)(**0**)(**0**)(**0.000448**)(**0.3417**) ;

slice Season*Canopy/sliceby=Canopy adjust=simulate lines;

slice Season*Canopy/sliceby=Season adjust=simulate lines;

Lsmeans Season*Canopy/ slice=Canopy lines mean cl adjust=simulate;

Lsmeans Season*Canopy/slice=Season lines mean cl adjust=simulate;

output out=Pred_butterflies_total_Richness Pred(ilink)=mu lcl(ilink)=lower ucl(ilink)=upper;

nloptions tech=NRRIDG Maxiter=**1000** maxfunc=**5000**;

run;

ods graphics on / imagename='Figure_total_Richness' width=**28** cm height=**26** cm;

ods listing image_dpi=**300**;

**proc** **sgpanel** data=Pred_butterflies_total_Richness noautolegend;

Panelby Season2 Canopy2 /novarname layout= lattice columns=**2** rows=**2** spacing=**2** uniscale=all sort=data

HEADERATTRS=(Color=black Family=Arial Size=**12** Style=normal Weight=normal);

Scatter x=elevation y=Sprichness / markerattrs=(symbol=circlefilled size=**20** color=coral) dataskin=Sheen name="name1" ;

Pbspline x=elevation y=mu / Lineattrs=(color=blue pattern=**1** thickness=**3**) nomarkers maxpoints=**100** nknots=**50** degree=**2** name="name2";

title height=**12** pt color=black "Richness of all butterfly families in cold and warm seasons and open and closed habitats";

colaxis label='Elevation (m)' Discreteorder=Data valuesrotate=diagonal LABELATTRS=(Color=black Family=Arial Size=**14**

Style=normal Weight=normal) VALUEATTRS=(Color=black Family=Arial Size=**10** Style=normal Weight=normal) ;

rowaxis label='Species richness' LABELATTRS=(Color=black Family=Arial Size=**12** Style=normal Weight=normal)

VALUEATTRS=(Color=black Family=Arial Size=**12** Style=normal Weight=normal);

*Keylegend "name1" "name2"/ Position=bottom title= '' autoitemsize VALUEATTRS=(family=Arial size=12) across=3 down=2 titleattrs=(color=black Family=Arial Size=12);

*colaxistable Count/Class=Habitat Position=bottom Separator valueattrs=(color=black family=Helvetica size=10)

LABELATTRS=(Color=black Family=Arial Size=11 Style=normal Weight=normal) /*title="Habitat type"*/ ;

/*valueattrs=(color=red)

labelattrs=(color=red)

titleattrs=(color=red);*/

**run**;

/*----Export abundance */

**PROC** **EXPORT** DATA=Classlevels_abund

OUTFILE= "D:\joseph_2009\Devolent\Butterflies\Results\Classlevels_abund.xlsx"

DBMS=EXCEL REPLACE;

SHEET="Classlevels_abund";

NEWFILE=YES;

**RUN**;

**PROC** **EXPORT** DATA=Diffs_abund

OUTFILE= "D:\joseph_2009\Devolent\Butterflies\Results\Diffs_abund.xlsx"

DBMS=EXCEL REPLACE;

SHEET="Diffs_abund";

NEWFILE=YES;

**RUN**;

**PROC** **EXPORT** DATA=Dimensions_abund

OUTFILE= "D:\joseph_2009\Devolent\Butterflies\Results\DiffDimensions_abund.xlsx"

DBMS=EXCEL REPLACE;

SHEET="Dimensions_abund";

NEWFILE=YES;

**RUN**;

**PROC** **EXPORT** DATA=Covparms_abund

OUTFILE= "D:\joseph_2009\Devolent\Butterflies\Results\Covparms_abund.xlsx"

DBMS=EXCEL REPLACE;

SHEET="Covparms_abund";

NEWFILE=YES;

**RUN**;

**PROC** **EXPORT** DATA=Lsmeans_abund

OUTFILE= "D:\joseph_2009\Devolent\Butterflies\Results\Lsmeans_abund.xlsx"

DBMS=EXCEL REPLACE;

SHEET="Lsmeans_abund";

NEWFILE=YES;

**RUN**;

**PROC** **EXPORT** DATA=Pred_butterflies_abund2

OUTFILE= "D:\joseph_2009\Devolent\Butterflies\Results\Pred_butterflies_abund.xlsx"

DBMS=EXCEL REPLACE;

SHEET="Pred_butterflies_abund";

NEWFILE=YES;

**RUN**;

**PROC** **EXPORT** DATA=Slicediffs_abund

OUTFILE= "D:\joseph_2009\Devolent\Butterflies\Results\Slicediffs_abund.xlsx"

DBMS=EXCEL REPLACE;

SHEET="Slicediffs_abund";

NEWFILE=YES;

**RUN**;

**PROC** **EXPORT** DATA=Slices_abund

OUTFILE= "D:\joseph_2009\Devolent\Butterflies\Results\Slices_abund.xlsx"

DBMS=EXCEL REPLACE;

SHEET="Slices_abund";

NEWFILE=YES;

**RUN**;

**PROC** **EXPORT** DATA=Tests1_abund

OUTFILE= "D:\joseph_2009\Devolent\Butterflies\Results\Tests1_abund.xlsx"

DBMS=EXCEL REPLACE;

SHEET="Tests1_abund";

NEWFILE=YES;

**RUN**;

**PROC** **EXPORT** DATA=Tests3_abund

OUTFILE= "D:\joseph_2009\Devolent\Butterflies\Results\Tests3_abund.xlsx"

DBMS=EXCEL REPLACE;

SHEET="Tests3_abund";

NEWFILE=YES;

**RUN**;

**PROC** **EXPORT** DATA=Parameterestimates_abund

OUTFILE= "D:\joseph_2009\Devolent\Butterflies\Results\Parameterestimates_abund.xlsx"

DBMS=EXCEL REPLACE;

SHEET="Parameterestimates_abund";

NEWFILE=YES;

**RUN**;

/*----Export total total abundance */

**PROC** **EXPORT** DATA=Classlevels_total_abund

OUTFILE= "D:\joseph_2009\Devolent\Butterflies\Results\Classlevels_total_abund.xlsx"

DBMS=EXCEL REPLACE;

SHEET="Classlevels_total_abund";

NEWFILE=YES;

**RUN**;

**PROC** **EXPORT** DATA=Diffs_total_abund

OUTFILE= "D:\joseph_2009\Devolent\Butterflies\Results\Diffs_total_abund.xlsx"

DBMS=EXCEL REPLACE;

SHEET="Diffs_total_abund";

NEWFILE=YES;

**RUN**;

**PROC** **EXPORT** DATA=Dimensions_total_abund

OUTFILE= "D:\joseph_2009\Devolent\Butterflies\Results\DiffDimensions_total_abund.xlsx"

DBMS=EXCEL REPLACE;

SHEET="Dimensions_total_abund";

NEWFILE=YES;

**RUN**;

**PROC** **EXPORT** DATA=Covparms_total_abund

OUTFILE= "D:\joseph_2009\Devolent\Butterflies\Results\Covparms_total_abund.xlsx"

DBMS=EXCEL REPLACE;

SHEET="Covparms_total_abund";

NEWFILE=YES;

**RUN**;

**PROC** **EXPORT** DATA=Lsmeans_total_abund

OUTFILE= "D:\joseph_2009\Devolent\Butterflies\Results\Lsmeans_total_abund.xlsx"

DBMS=EXCEL REPLACE;

SHEET="Lsmeans_total_abund";

NEWFILE=YES;

**RUN**;

**PROC** **EXPORT** DATA=Pred_butterflies_total_abund

OUTFILE= "D:\joseph_2009\Devolent\Butterflies\Results\Pred_butterflies_total_abund.xlsx"

DBMS=EXCEL REPLACE;

SHEET="Pred_butterflies_total_abund";

NEWFILE=YES;

**RUN**;

**PROC** **EXPORT** DATA=Slicediffs_total_abund

OUTFILE= "D:\joseph_2009\Devolent\Butterflies\Results\Slicediffs_total_abund.xlsx"

DBMS=EXCEL REPLACE;

SHEET="Slicediffs_total_abund";

NEWFILE=YES;

**RUN**;

**PROC** **EXPORT** DATA=Slices_total_abund

OUTFILE= "D:\joseph_2009\Devolent\Butterflies\Results\Slices_total_abund.xlsx"

DBMS=EXCEL REPLACE;

SHEET="Slices_total_abund";

NEWFILE=YES;

**RUN**;

**PROC** **EXPORT** DATA=Tests1_total_abund

OUTFILE= "D:\joseph_2009\Devolent\Butterflies\Results\Tests1_total_abund.xlsx"

DBMS=EXCEL REPLACE;

SHEET="Tests1_total_abund";

NEWFILE=YES;

**RUN**;

**PROC** **EXPORT** DATA=Tests3_total_abund

OUTFILE= "D:\joseph_2009\Devolent\Butterflies\Results\Tests3_total_abund.xlsx"

DBMS=EXCEL REPLACE;

SHEET="Tests3_total_abund";

NEWFILE=YES;

**RUN**;

**PROC** **EXPORT** DATA=Parameterestimates_total_abund

OUTFILE= "D:\joseph_2009\Devolent\Butterflies\Results\Parameterestimates_total_abund.xlsx"

DBMS=EXCEL REPLACE;

SHEET="Parameterestimates_total_abund";

NEWFILE=YES;

**RUN**;

/*----Export richness */

**PROC** **EXPORT** DATA=Classlevels_richness

OUTFILE= "D:\joseph_2009\Devolent\Butterflies\Results\Classlevels_richness.xlsx"

DBMS=EXCEL REPLACE;

SHEET="Classlevels_richness";

NEWFILE=YES;

**RUN**;

**PROC** **EXPORT** DATA=Diffs_richness

OUTFILE= "D:\joseph_2009\Devolent\Butterflies\Results\Diffs_richness.xlsx"

DBMS=EXCEL REPLACE;

SHEET="Diffs_richness";

NEWFILE=YES;

**RUN**;

**PROC** **EXPORT** DATA=Dimensions_richness

OUTFILE= "D:\joseph_2009\Devolent\Butterflies\Results\DiffDimensions_total_richness.xlsx"

DBMS=EXCEL REPLACE;

SHEET="Dimensions_richness";

NEWFILE=YES;

**RUN**;

**PROC** **EXPORT** DATA=Covparms_richness

OUTFILE= "D:\joseph_2009\Devolent\Butterflies\Results\Covparms_richness.xlsx"

DBMS=EXCEL REPLACE;

SHEET="Covparms_richness";

NEWFILE=YES;

**RUN**;

**PROC** **EXPORT** DATA=Lsmeans_richness

OUTFILE= "D:\joseph_2009\Devolent\Butterflies\Results\Lsmeans_richness.xlsx"

DBMS=EXCEL REPLACE;

SHEET="Lsmeans_richness";

NEWFILE=YES;

**RUN**;

**PROC** **EXPORT** DATA=Pred_butterflies_rich2

OUTFILE= "D:\joseph_2009\Devolent\Butterflies\Results\Pred_butterflies_rich2.xlsx"

DBMS=EXCEL REPLACE;

SHEET="Pred_butterflies_rich2";

NEWFILE=YES;

**RUN**;

**PROC** **EXPORT** DATA=Slicediffs_rich

OUTFILE= "D:\joseph_2009\Devolent\Butterflies\Results\Slicediffs_richn.xlsx"

DBMS=EXCEL REPLACE;

SHEET="Slicediffs_rich";

NEWFILE=YES;

**RUN**;

**PROC** **EXPORT** DATA=Slices_rich

OUTFILE= "D:\joseph_2009\Devolent\Butterflies\Results\Slices_rich.xlsx"

DBMS=EXCEL REPLACE;

SHEET="Slices_rich";

NEWFILE=YES;

**RUN**;

**PROC** **EXPORT** DATA=Tests1_rich

OUTFILE= "D:\joseph_2009\Devolent\Butterflies\Results\Tests1_rich.xlsx"

DBMS=EXCEL REPLACE;

SHEET="Tests1_rich";

NEWFILE=YES;

**RUN**;

**PROC** **EXPORT** DATA=Tests3_rich

OUTFILE= "D:\joseph_2009\Devolent\Butterflies\Results\Tests3_rich.xlsx"

DBMS=EXCEL REPLACE;

SHEET="Tests3_rich";

NEWFILE=YES;

**RUN**;

**PROC** **EXPORT** DATA=Parameterestimates_rich

OUTFILE= "D:\joseph_2009\Devolent\Butterflies\Results\Parameterestimates_rich.xlsx"

DBMS=EXCEL REPLACE;

SHEET="Parameterestimates_rich";

NEWFILE=YES;

**RUN**;

/***Export total richness----*/

**PROC** **EXPORT** DATA=Classlevels_total_richness

OUTFILE= "D:\joseph_2009\Devolent\Butterflies\Results\Classlevels_total_richness.xlsx"

DBMS=EXCEL REPLACE;

SHEET="Classlevels_total_richness";

NEWFILE=YES;

**RUN**;

**PROC** **EXPORT** DATA=Diffs_total_richness

OUTFILE= "D:\joseph_2009\Devolent\Butterflies\Results\Diffs_total_richness.xlsx"

DBMS=EXCEL REPLACE;

SHEET="Diffs_total_richness";

NEWFILE=YES;

**RUN**;

**PROC** **EXPORT** DATA=Dimensions_total_richness

OUTFILE= "D:\joseph_2009\Devolent\Butterflies\Results\DiffDimensions_total_richness.xlsx"

DBMS=EXCEL REPLACE;

SHEET="Dimensions_total_richness";

NEWFILE=YES;

**RUN**;

**PROC** **EXPORT** DATA=Covparms_total_richness

OUTFILE= "D:\joseph_2009\Devolent\Butterflies\Results\Covparms_total_richness.xlsx"

DBMS=EXCEL REPLACE;

SHEET="Covparms_total_richness";

NEWFILE=YES;

**RUN**;

**PROC** **EXPORT** DATA=Lsmeans_total_richness

OUTFILE= "D:\joseph_2009\Devolent\Butterflies\Results\Lsmeans_total_richness.xlsx"

DBMS=EXCEL REPLACE;

SHEET="Lsmeans_total_richness";

NEWFILE=YES;

**RUN**;

**PROC** **EXPORT** DATA=Pred_butterflies_total_richness

OUTFILE= "D:\joseph_2009\Devolent\Butterflies\Results\Pred_butterflies_total_richness.xlsx"

DBMS=EXCEL REPLACE;

SHEET="Pred_butterflies_total_richness";

NEWFILE=YES;

**RUN**;

**PROC** **EXPORT** DATA=Slicediffs_total_richness

OUTFILE= "D:\joseph_2009\Devolent\Butterflies\Results\Slicediffs_total_richness.xlsx"

DBMS=EXCEL REPLACE;

SHEET="Slicediffs_total_richness";

NEWFILE=YES;

**RUN**;

**PROC** **EXPORT** DATA=Slices_total_richness

OUTFILE= "D:\joseph_2009\Devolent\Butterflies\Results\Slices_total_richness.xlsx"

DBMS=EXCEL REPLACE;

SHEET="Slices_total_richness";

NEWFILE=YES;

**RUN**;

**PROC** **EXPORT** DATA=Tests1_total_richness

OUTFILE= "D:\joseph_2009\Devolent\Butterflies\Results\Tests1_total_richness.xlsx"

DBMS=EXCEL REPLACE;

SHEET="Tests1_total_richness";

NEWFILE=YES;

**RUN**;

**PROC** **EXPORT** DATA=Tests3_total_richness

OUTFILE= "D:\joseph_2009\Devolent\Butterflies\Results\Tests3_total_richness.xlsx"

DBMS=EXCEL REPLACE;

SHEET="Tests3_total_richness";

NEWFILE=YES;

**RUN**;

**PROC** **EXPORT** DATA=Parameterestimates_total_rich

OUTFILE= "D:\joseph_2009\Devolent\Butterflies\Results\Parameterestimates_total_rich.xlsx"

DBMS=EXCEL REPLACE;

SHEET="Parameterestimates_total_rich";

NEWFILE=YES;

**RUN**;
