# Supplementary material for: Elevational distribution of montane Afrotropical butterflies is influenced by seasonality and habitat structure
Source: PLoS One. 2022 Jul 5;17(7):e0270769. doi: 10.1371/journal.pone.0270769 (PMC9255748; doi:10.1371/journal.pone.0270769)
Supplement: S1 Table — (DOCX) [file pone.0270769.s003.docx]

**S1 Table.** Description of sampling locations along two elevational transects in the Uluguru Mountains.

| **Transect location** | **Sampling location** | **GPS coordinates** | | | **Elevation (m)** | **Canopy closure (%)** | **Habitat structure** |
| --- | --- | --- | --- | --- | --- | --- | --- |
|  |  | **Latitude** | | **Longitude** |  |  |  |
| Bondwa | B-S1 | -6.8948 | 37.6713 | | 1542 | 0.16 | open |
| Bondwa | B-S2 | -6.8958 | 37.6709 | | 1582 | 96.10 | closed |
| Bondwa | B-S3 | -6.8964 | 37.6706 | | 1626 | 48.26 | open |
| Bondwa | B-S4 | -6.8971 | 37.6705 | | 1663 ⃰ | 97.92 | closed |
| Bondwa | B-S5 | -6.8979 | 37.6704 | | 1703 | 94.02 | closed |
| Bondwa | B-S6 | -6.8980 | 37.6716 | | 1752 | 85.44 | closed |
| Bondwa | B-S7 | -6.8984 | 37.6719 | | 1766 | 90.64 | closed |
| Bondwa | B-S8 | -6.8993 | 37.6716 | | 1836 ⃰ | 81.80 | closed |
| Bondwa | B-S9 | -6.9006 | 37.6718 | | 1885 | 93.24 | closed |
| Bondwa | B-S10 | -6.9013 | 37.6716 | | 1922 | 97.14 | closed |
| Bondwa | B-S11 | -6.9024 | 37.6708 | | 1971 | 91.16 | closed |
| Bondwa | B-S12 | -6.9030 | 37.6709 | | 2004 ⃰ | 96.10 | closed |
| Bondwa | B-S13 | -6.9036 | 37.6715 | | 2045 | 92.46 | closed |
| Bondwa | B-S14 | -6.9038 | 37.6722 | | 2091 | 83.88 | closed |
| Bondwa | B-S15 | -6.9050 | 37.6736 | | 2133 | 86.74 | closed |
| Bondwa | B-S16 | -6.9055 | 37.6732 | | 2159 ⃰ | 34.74 | open |
| Lukwangule | L-S17 | -7.1105 | 37.6037 | | 2206 | 70.62 | closed |
| Lukwangule | L-S18 | -7.1106 | 37.6049 | | 2248 | 72.44 | closed |
| Lukwangule | L-S19 | -7.1117 | 37.6072 | | 2297 | 81.28 | closed |
| Lukwangule | L-S20 | -7.1135 | 37.6088 | | 2351 | 83.88 | closed |
| Lukwangule | L-S21 | -7.1139 | 37.6094 | | 2383 | 91.94 | closed |
| Lukwangule | L-S22 | -7.1145 | 37.6099 | | 2416 | 85.70 | closed |
| Lukwangule | L-S23 | -7.1153 | 37.6108 | | 2462 | 89.08 | closed |
| Lukwangule | L-S24 | -7.1150 | 37.6119 | | 2492 | 5.36 | open |
| Lukwangule | L-S25 | -7.1131 | 37.6138 | | 2541 | 72.70 | closed |
| Lukwangule | L-S26 | -7.0980 | 37.6158 | | 2588 ⃰ | 0.16 | open |
| Lukwangule | L-S27 | -7.0936 | 37.6191 | | 2604 | 0.16 | open |
| Lukwangule | L-S28 | -7.0910 | 37.6209 | | 2639 | 0.16 | open |

* Elevational location of a temperature logger.

[Note: B = Bondwa, L = Lukwangule and S = Site]
