# Supplementary material for: Elevational distribution of montane Afrotropical butterflies is influenced by seasonality and habitat structure
Source: PLoS One. 2022 Jul 5;17(7):e0270769. doi: 10.1371/journal.pone.0270769 (PMC9255748; doi:10.1371/journal.pone.0270769)
Supplement: S6 Table — (DOCX) [file pone.0270769.s008.docx]

**S6 Table.** Parameter estimates and associated standard errors and 95% confidence intervals for the semi-parametric generalized linear mixed models for (a) species richness, (b) species richness by family, (c) abundance, and (d) abundance by family.

1. **Species richness**

| **Effect** | **Season** | **Habitat structure** | **Estimate** | **StdErr** | **DF** | **tValue** | **Probt** | **Alpha** | **Lower 95% CL** | **Upper 95% CL** |
| --- | --- | --- | --- | --- | --- | --- | --- | --- | --- | --- |
| Season | long to short rains transition |  | 4.085 | 3.195 | 11.176 | 1.280 | 0.226 | 0.05 | -2.924 | 11.094 |
| Season | short to long rains transition |  | 9.639 | 2.317 | 8.434 | 4.159 | 0.003 | 0.05 | 4.342 | 14.935 |
| Habitat structure |  | Closed | -1.076 | 1.103 | 48 | -0.976 | 0.334 | 0.05 | -3.294 | 1.141 |
| Habitat structure |  | Open | 0 |  |  |  |  |  |  |  |
| Season*Habitat structure | long to short rains transition | Closed | -1.129 | 1.770 | 48 | -0.638 | 0.526 | 0.05 | -4.689 | 2.430 |
| Season*Habitat structure | long to short rains transition | Open | 0 |  |  |  |  |  |  |  |
| Season*Habitat structure | short to long rains transition | Closed | 0 |  |  |  |  |  |  |  |
| Season*Habitat structure | short to long rains transition | Open | 0 |  |  |  |  |  |  |  |
| Elevation*Season*Habitat structure | long to short rains transition | Closed | -0.001 | 0.001 | 11.464 | -0.411 | 0.689 | 0.05 | -0.004 | 0.003 |
| Elevation*Season*Habitat structure | long to short rains transition | Open | -0.001 | 0.001 | 12.254 | -0.802 | 0.438 | 0.05 | -0.004 | 0.002 |
| Elevation*Season*Habitat structure | short to long rains transition | Closed | -0.003 | 0.001 | 8.060 | -2.862 | 0.021 | 0.05 | -0.006 | -0.001 |
| Elevation*Season*Habitat structure | short to long rains transition | Open | -0.003 | 0.001 | 9.496 | -3.162 | 0.011 | 0.05 | -0.005 | -0.001 |

1. **Species richness by family**

| **Effect** | **Season** | | **Family** | **Habitat structure** | **Estimate** | **StdErr** | **DF** | **tValue** | **Probt** | **Alpha** | Lower 95%CL | Upper 95%CL |
| --- | --- | --- | --- | --- | --- | --- | --- | --- | --- | --- | --- | --- |
| Season | long to short rains trans. |  | |  | 1.702 | 6.604 | 25.129 | 0.258 | 0.799 | 0.05 | -11.896 | 15.299 |
| Season | short to long rains trans. |  | |  | 1.625 | 5.025 | 23.656 | 0.323 | 0.749 | 0.05 | -8.754 | 12.004 |
| Habitat structure |  |  | | Closed | -4.373 | 2.748 | 144 | -1.591 | 0.114 | 0.05 | -9.805 | 1.059 |
| Habitat structure |  |  | | Open | 0 |  |  |  |  |  |  |  |
| Season*Habitat structure | long to short rains trans. |  | | Closed | -8.479 | 6.032 | 144 | -1.406 | 0.162 | 0.05 | -20.402 | 3.444 |
| Season*Habitat structure | long to short rains trans. |  | | Open | 0 |  |  |  |  |  |  |  |
| Season*Habitat structure | short to long rains trans. |  | | Closed | 0 |  |  |  |  |  |  |  |
| Season*Habitat structure | short to long rains trans. |  | | Open | 0 |  |  |  |  |  |  |  |
| Family |  | Nymphalidae | |  | 6.723 | 6.330 | 11.958 | 1.062 | 0.309 | 0.05 | -7.074 | 20.521 |
| Family |  | Papilionidae | |  | 29.977 | 10.496 | 14.930 | 2.856 | 0.012 | 0.05 | 7.596 | 52.359 |
| Family |  | Pieridae | |  | 0 |  |  |  |  |  |  |  |
| Season*Family | long to short rains trans. | Nymphalidae | |  | -8.326 | 8.800 | 10.200 | -0.946 | 0.366 | 0.05 | -27.881 | 11.229 |
| Season*Family | long to short rains trans. | Papilionidae | |  | -1.322 | 13.245 | 5.891 | -0.100 | 0.924 | 0.05 | -33.876 | 31.231 |
| Season*Family | long to short rains trans. | Pieridae | |  | 0 |  |  |  |  |  |  |  |
| Season*Family | short to long rains trans. | Nymphalidae | |  | 0 |  |  |  |  |  |  |  |
| Season*Family | short to long rains trans. | Papilionidae | |  | 0 |  |  |  |  |  |  |  |
| Season*Family | short to long rains trans. | Pieridae | |  | 0 |  |  |  |  |  |  |  |
| Family*Habitat structure |  | Nymphalidae | | Closed | 2.797 | 3.237 | 144 | 0.864 | 0.389 | 0.05 | -3.601 | 9.196 |
| Family*Habitat structure |  | Nymphalidae | | Open | 0 |  |  |  |  |  |  |  |
| Family*Habitat structure |  | Papilionidae | | Closed | 2.754 | 3.912 | 144 | 0.704 | 0.483 | 0.05 | -4.978 | 10.486 |
| Family*Habitat structure |  | Papilionidae | | Open | 0 |  |  |  |  |  |  |  |
| Family*Habitat structure |  | Pieridae | | Closed | 0 |  |  |  |  |  |  |  |
| Family*Habitat structure |  | Pieridae | | Open | 0 |  |  |  |  |  |  |  |
| Season*Family*Habitat structure | long to short rains trans. | Nymphalidae | | Closed | 10.024 | 6.510 | 144 | 1.540 | 0.126 | 0.05 | -2.842 | 22.891 |
| Season*Family*Habitat structure | long to short rains trans. | Nymphalidae | | Open | 0 |  |  |  |  |  |  |  |
| Season*Family*Habitat structure | long to short rains trans. | Papilionidae | | Closed | 9.661 | 8.467 | 144 | 1.141 | 0.256 | 0.05 | -7.074 | 26.396 |
| Season*Family*Habitat structure | long to short rains trans. | Papilionidae | | Open | 0 |  |  |  |  |  |  |  |
| Season*Family*Habitat structure | long to short rains trans. | Pieridae | | Closed | 0 |  |  |  |  |  |  |  |
| Season*Family*Habitat structure | long to short rains trans. | Pieridae | | Open | 0 |  |  |  |  |  |  |  |
| Season*Family*Habitat structure | short to long rains trans. | Nymphalidae | | Closed | 0 |  |  |  |  |  |  |  |
| Season*Family*Habitat structure | short to long rains trans. | Nymphalidae | | Open | 0 |  |  |  |  |  |  |  |
| Season*Family*Habitat structure | short to long rains trans. | Papilionidae | | Closed | 0 |  |  |  |  |  |  |  |
| Season*Family*Habitat structure | short to long rains trans. | Papilionidae | | Open | 0 |  |  |  |  |  |  |  |
| Season*Family*Habitat structure | short to long rains trans. | Pieridae | | Closed | 0 |  |  |  |  |  |  |  |
| Season*Family*Habitat structure | short to long rains trans. | Pieridae | | Open | 0 |  |  |  |  |  |  |  |
| Elevation*Season*Family*Habitat structure | long to short rains trans. | Nymphalidae | | Closed | -0.0005 | 0.003 | 25.377 | -0.179 | 0.859 | 0.05 | -0.006 | 0.005 |
| Elevation*Season*Family*Habitat structure | long to short rains trans. | Nymphalidae | | Open | 0.0003 | 0.002 | 19.722 | 0.133 | 0.896 | 0.05 | -0.004 | 0.005 |
| Elevation*Season*Family*Habitat structure | long to short rains trans. | Papilionidae | | Closed | -0.014 | 0.005 | 20.666 | -2.846 | 0.010 | 0.05 | -0.024 | -0.004 |
| Elevation*Season*Family*Habitat structure | long to short rains trans. | Papilionidae | | Open | -0.013 | 0.005 | 21.188 | -2.639 | 0.0153 | 0.05 | -0.023 | -0.003 |
| Elevation*Season*Family*Habitat structure | long to short rains trans. | Pieridae | | Closed | 0.0047 | 0.003 | 44.822 | 1.754 | 0.086 | 0.05 | -0.001 | 0.010 |
| Elevation*Season*Family*Habitat structure | long to short rains trans. | Pieridae | | Open | -0.005 | 0.003 | 26.522 | -0.181 | 0.858 | 0.05 | -0.006 | 0.005 |
| Elevation*Season*Family*Habitat structure | short to long rains trans. | Nymphalidae | | Closed | -0.003 | 0.002 | 15.207 | -1.399 | 0.182 | 0.05 | -0.007 | 0.001 |
| Elevation*Season*Family*Habitat structure | short to long rains trans. | Nymphalidae | | Open | -0.003 | 0.002 | 13.886 | -1.638 | 0.124 | 0.05 | -0.006 | 0.001 |
| Elevation*Season*Family*Habitat structure | short to long rains trans. | Papilionidae | | Closed | -0.013 | 0.004 | 16.395 | -3.279 | 0.005 | 0.05 | -0.022 | -0.005 |
| Elevation*Season*Family*Habitat structure | short to long rains trans. | Papilionidae | | Open | -0.013 | 0.004 | 21.963 | -3.220 | 0.004 | 0.05 | -0.022 | -0.005 |
| Elevation*Season*Family*Habitat structure | short to long rains trans. | Pieridae | | Closed | 0.001 | 0.002 | 16.787 | 0.578 | 0.570 | 0.05 | -0.003 | 0.006 |
| Elevation*Season*Family*Habitat structure | short to long rains trans. | Pieridae | | Open | -0.0002 | 0.002 | 25.297 | -0.087 | 0.931 | 0.05 | -0.004 | 0.004 |

1. **Abundance**

| **Effect** | **Season** | **Habitat structure** | **Estimate** | **StdErr** | **DF** | **tValue** | **Probt** | **Alpha** | **Lower 95%CL** | **Upper 95% CL** |
| --- | --- | --- | --- | --- | --- | --- | --- | --- | --- | --- |
| Season | long to short rains transition |  | 6.019 | 4.736 | 3.993 | 1.271 | 0.273 | 0.05 | -7.139 | 19.177 |
| Season | short to long rains transition |  | 5.056 | 4.646 | 4.232 | 1.088 | 0.335 | 0.05 | -7.568 | 17.680 |
| Habitat structure |  | Closed | -1.385 | 6.046 | 4.493 | -0.229 | 0.829 | 0.05 | -17.470 | 14.700 |
| Habitat structure |  | Open | 0 |  |  |  |  |  |  |  |
| Season*Habitat structure | long to short rains transition | Closed | -6.691 | 8.685 | 4.369 | -0.770 | 0.481 | 0.05 | -30.024 | 16.641 |
| Season*Habitat structure | long to short rains transition | Open | 0 |  |  |  |  |  |  |  |
| Season*Habitat structure | short to long rains transition | Closed | 0 |  |  |  |  |  |  |  |
| Season*Habitat structure | short to long rains transition | Open | 0 |  |  |  |  |  |  |  |
| Elevation*Season*Habitat structure | long to short rains transition | Closed | 0.002 | 0.002 | 5.127 | 0.985 | 0.369 | 0.05 | -0.003 | 0.006 |
| Elevation*Season*Habitat structure | long to short rains transition | Open | -0.001 | 0.002 | 4.255 | -0.683 | 0.530 | 0.05 | -0.006 | 0.004 |
| Elevation*Season*Habitat structure | short to long rains transition | Closed | -0.0003 | 0.002 | 5.394 | -0.181 | 0.863 | 0.05 | -0.004 | 0.004 |
| Elevation*Season*Habitat structure | short to long rains transition | Open | -0.0002 | 0.002 | 4.486 | -0.123 | 0.908 | 0.05 | -0.005 | 0.005 |

1. **Abundance by family**

| **Effect** | **Season** | **Family** | **Habitat structure** | **Estimate** | **StdErr** | **DF** | **tValue** | **Probt** | **Alpha** | **Lower 95% CL** | **Upper 95% CL** |
| --- | --- | --- | --- | --- | --- | --- | --- | --- | --- | --- | --- |
| Season | long to short rains transition |  |  | -5.710 | 10.706 | 16.519 | -0.533 | 0.601 | 0.05 | -28.348 | 16.927 |
| Season | short to long rains transition |  |  | -4.799 | 10.536 | 14.657 | -0.456 | 0.655 | 0.05 | -27.302 | 17.704 |
| Habitat structure |  |  | Closed | 10.567 | 11.577 | 4.807 | 0.913 | 0.405 | 0.05 | -19.556 | 40.691 |
| Habitat structure |  |  | Open | 0 |  |  |  |  |  |  |  |
| Season*Habitat structure | long to short rains transition |  | Closed | -7.503 | 6.594 | 47.964 | -1.138 | 0.261 | 0.05 | -20.761 | 5.755 |
| Season*Habitat structure | long to short rains transition |  | Open | 0 |  |  |  |  |  |  |  |
| Season*Habitat structure | short to long rains transition |  | Closed | 0 |  |  |  |  |  |  |  |
| Season*Habitat structure | short to long rains transition |  | Open | 0 |  |  |  |  |  |  |  |
| Family |  | Nymphalidae | | 6.177 | 14.584 | 13.394 | 0.424 | 0.679 | 0.05 | -25.235 | 37.589 |
| Family |  | Papilionidae | | 74.686 | 35.870 | 6.637 | 2.082 | 0.078 | 0.05 | -11.084 | 160.456 |
| Family |  | Pieridae |  | 0 |  |  |  |  |  |  |  |
| Season*Family | long to short rains transition | Nymphalidae | | -3.551 | 11.955 | 2.503 | -0.297 | 0.789 | 0.05 | -46.249 | 39.147 |
| Season*Family | long to short rains transition | Papilionidae | | -3.096 | 15.094 | 1.629 | -0.205 | 0.860 | 0.05 | -84.498 | 78.306 |
| Season*Family | long to short rains transition | Pieridae |  | 0 |  |  |  |  |  |  |  |
| Season*Family | short to long rains transition | Nymphalidae | | 0 |  |  |  |  |  |  |  |
| Season*Family | short to long rains transition | Papilionidae | | 0 |  |  |  |  |  |  |  |
| Season*Family | short to long rains transition | Pieridae |  | 0 |  |  |  |  |  |  |  |
| Family*Habitat structure | | Nymphalidae | Closed | -26.138 | 16.097 | 4.294 | -1.624 | 0.175 | 0.05 | -69.647 | 17.372 |
| Family*Habitat structure | | Nymphalidae | Open | 0 |  |  |  |  |  |  |  |
| Family*Habitat structure | | Papilionidae | Closed | -25.350 | 35.973 | 1.752 | -0.705 | 0.563 | 0.05 | -203.094 | 152.394 |
| Family*Habitat structure | | Papilionidae | Open | 0 |  |  |  |  |  |  |  |
| Family*Habitat structure | | Pieridae | Closed | 0 |  |  |  |  |  |  |  |
| Family*Habitat structure | | Pieridae | Open | 0 |  |  |  |  |  |  |  |
| Season*Family*Habitat structure | long to short rains transition | Nymphalidae | Closed | 9.669 | 7.431 | 64.125 | 1.301 | 0.198 | 0.05 | -5.176 | 24.515 |
| Season*Family*Habitat structure | long to short rains transition | Nymphalidae | Open | 0 |  |  |  |  |  |  |  |
| Season*Family*Habitat structure | long to short rains transition | Papilionidae | Closed | 3.407 | 10.010 | 82.382 | 0.340 | 0.734 | 0.05 | -16.504 | 23.318 |
| Season*Family*Habitat structure | long to short rains transition | Papilionidae | Open | 0 |  |  |  |  |  |  |  |
| Season*Family*Habitat structure | long to short rains transition | Pieridae | Closed | 0 |  |  |  |  |  |  |  |
| Season*Family*Habitat structure | long to short rains transition | Pieridae | Open | 0 |  |  |  |  |  |  |  |
| Season*Family*Habitat structure | short to long rains transition | Nymphalidae | Closed | 0 |  |  |  |  |  |  |  |
| Season*Family*Habitat structure | short to long rains transition | Nymphalidae | Open | 0 |  |  |  |  |  |  |  |
| Season*Family*Habitat structure | short to long rains transition | Papilionidae | Closed | 0 |  |  |  |  |  |  |  |
| Season*Family*Habitat structure | short to long rains transition | Papilionidae | Open | 0 |  |  |  |  |  |  |  |
| Season*Family*Habitat structure | short to long rains transition | Pieridae | Closed | 0 |  |  |  |  |  |  |  |
| Season*Family*Habitat structure | short to long rains transition | Pieridae | Open | 0 |  |  |  |  |  |  |  |
| Elevation*Season*Family*Habitat structure | long to short rains transition | Nymphalidae | Closed | 0.006 | 0.006 | 11.154 | 1.031 | 0.325 | 0.05 | -0.007 | 0.020 |
| Elevation*Season*Family*Habitat structure | long to short rains transition | Nymphalidae | Open | 0.002 | 0.004 | 13.315 | 0.428 | 0.675 | 0.05 | -0.007 | 0.011 |
| Elevation*Season*Family*Habitat structure | long to short rains transition | Papilionidae | Closed | -0.021 | 0.010 | 5.854 | -2.156 | 0.076 | 0.05 | -0.044 | 0.003 |
| Elevation*Season*Family*Habitat structure | long to short rains transition | Papilionidae | Open | -0.028 | 0.015 | 6.734 | -1.858 | 0.107 | 0.05 | -0.064 | 0.008 |
| Elevation*Season*Family*Habitat structure | long to short rains transition | Pieridae | Closed | 0.002 | 0.005 | 12.081 | 0.440 | 0.668 | 0.05 | -0.008 | 0.013 |
| Elevation*Season*Family*Habitat structure | long to short rains transition | Pieridae | Open | 0.003 | 0.004 | 17.131 | 0.725 | 0.479 | 0.05 | -0.006 | 0.012 |
| Elevation*Season*Family*Habitat structure | short to long rains transition | Nymphalidae | Closed | 0.006 | 0.005 | 9.541 | 1.114 | 0.292 | 0.05 | -0.006 | 0.0184 |
| Elevation*Season*Family*Habitat structure | short to long rains transition | Nymphalidae | Open | 0.0004 | 0.004 | 12.761 | 0.103 | 0.920 | 0.05 | -0.008 | 0.009 |
| Elevation*Season*Family*Habitat structure | short to long rains transition | Papilionidae | Closed | -0.024 | 0.009 | 5.622 | -2.526 | 0.0475 | 0.05 | -0.048 | -0.0003 |
| Elevation*Season*Family*Habitat structure | short to long rains transition | Papilionidae | Open | -0.029 | 0.015 | 6.050 | -1.944 | 0.100 | 0.05 | -0.066 | 0.008 |
| Elevation*Season*Family*Habitat structure | short to long rains transition | Pieridae | Closed | -0.001 | 0.005 | 11.684 | -0.233 | 0.820 | 0.05 | -0.012 | 0.010 |
| Elevation*Season*Family*Habitat structure | short to long rains transition | Pieridae | Open | 0.004 | 0.004 | 15.279 | 0.855 | 0.406 | 0.05 | -0.005 | 0.012 |
