# Supplementary figures and images for: Elevational distribution of montane Afrotropical butterflies is influenced by seasonality and habitat structure
Source: PLoS One. 2022 Jul 5;17(7):e0270769. doi: 10.1371/journal.pone.0270769 (PMC9255748; doi:10.1371/journal.pone.0270769)

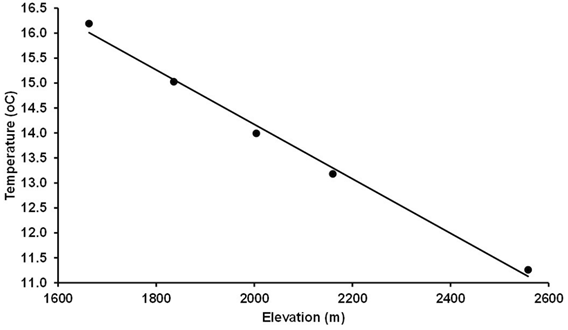

Supplement: S1 Fig — Air temperature was recorded with loggers at 1-h intervals at 1663, 1836, 2004, 2159, and 2588 m over a 14-month period between August 2019 and September 2020. The straight line is described by the following equation: y = 25.1–0.0055 (x). (TIF) [file pone.0270769.s009.tif]

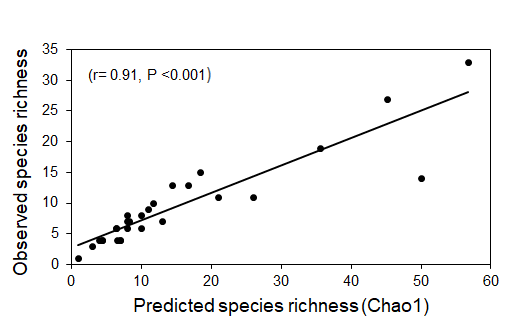

Supplement: S2 Fig — Predicted species richness was calculated with the abundance-based bias-corrected Chao1 estimator. (TIF) [file pone.0270769.s010.tif]
